# Supplementary material for: Risperidone versus placebo for aggression following traumatic brain injury: a feasibility randomised controlled trial
Source: BMJ Open. 2020 Sep 10;10(9):e036300. doi: 10.1136/bmjopen-2019-036300 (PMC7485257; doi:10.1136/bmjopen-2019-036300)
Supplement: Author's manuscript [file bmjopen-2019-036300.draft_revisions.pdf]

BMJ Open

BMJ Open is committed to open peer review. As part of this commitment we make the peer review history of every article we publish publicly available.

When an article is published we post the peer reviewers' comments and the authors' responses online. We also post the versions of the paper that were used during peer review. These are the versions that the peer review comments apply to.

The versions of the paper that follow are the versions that were submitted during the peer review process. They are not the versions of record or the final published versions. They should not be cited or distributed as the published version of this manuscript.

BMJ Open is an open access journal and the full, final, typeset and author-corrected version of record of the manuscript is available on our site with no access controls, subscription charges or pay-per-view fees (http://bmjopen.bmj.com).

If you have any questions on BMJ Open's open peer review process please email info.bmjopen@bmj.com

BMJ Open

Aggression Following Traumatic Brain Injury: Effectiveness of Risperidone (AFTER)-a feasibility RCT.

Journal:	*BMJ Open*
Manuscript ID	bmjopen-2019-036300
Article Type:	Original research
Date Submitted by the Author:	10-Dec-2019

Complete List of Authors:	Deb, Shoumitro (Shoumi); Imperial College London, Centre for Psychiatry<br>Aimola, Lina; Imperial College London, Division of Brain Sciences<br>Leeson, Verity; Imperial College London<br>Bodani, Mayur; Kent and Medway NHS and Social Care Partnership Trust<br>Li, Lucia; Imperial College London<br>Weaver, Tim ; Middlesex University<br>Sharp, David; Imperial College London, Division of Brain Sciences<br>Bassett, Paul; Statsconsultancy Ltd,<br>Crawford, Mike; Imperial College London, Psychological Medicine

Keywords:	PSYCHIATRY, Adult psychiatry < PSYCHIATRY, Impulse control disorders < PSYCHIATRY

SCHOLARONE™ Manuscripts

I, the Submitting Author has the right to grant and does grant on behalf of all authors of the Work (as defined in the below author licence), an exclusive licence and/or a non-exclusive licence for contributions from authors who are: i) UK Crown employees; ii) where BMJ has agreed a CC-BY licence shall apply, and/or iii) in accordance with the terms applicable for US Federal Government officers or employees acting as part of their official duties; on a worldwide, perpetual, irrevocable, royalty-free basis to BMJ Publishing Group Ltd ("BMJ") its licensees and where the relevant Journal is co-owned by BMJ to the co-owners of the Journal, to publish the Work in this journal and any other BMJ products and to exploit all rights, as set out in our licence.

The Submitting Author accepts and understands that any supply made under these terms is made by BMJ to the Submitting Author unless you are acting as an employee on behalf of your employer or a postgraduate student of an affiliated institution which is paying any applicable article publishing charge ("APC") for Open Access articles. Where the Submitting Author wishes to make the Work available on an Open Access basis (and intends to pay the relevant APC), the terms of reuse of such Open Access shall be governed by a Creative Commons licence – details of these licences and which Creative Commons licence will apply to this Work are set out in our licence referred to above.

Other than as permitted in any relevant BMJ Author's Self Archiving Policies, I confirm this Work has not been accepted for publication elsewhere, is not being considered for publication elsewhere and does not duplicate material already published. I confirm all authors consent to publication of this Work and authorise the granting of this licence.

For peer review only

Title

Aggression Following Traumatic Brain Injury: Effectiveness of Risperidone (*AFTER*)-a feasibility RCT.

Authors

Shoumitro Deb,[1] Lina Aimola,[1] Verity Leeson,[1] Mayur Bodani,[2] Lucia Li,[1] Tim Weaver,[3] David Sharp,[1] Paul Bassett,[4] Mike Crawford.[1]

[1]Division on Brain Sciences, Imperial College London; [2]Kent and Medway NHS and Social Care Partnership NHS Trust, [3]Middlesex University.

Correspondence to

Professor Shoumitro (Shoumi) Deb;

s.deb@imperial.ac.uk

Abstract

Objectives To conduct a feasibility RCT of risperidone for the treatment of aggression in adults with traumatic brain injury (TBI) to assess feasibility for a substantive full scale definitive RCT in future.

Design Multi-centre parallel design placebo controlled (1:1 ratio) double-blind feasibility RCT with an embedded process evaluation.

Setting Four psychiatric and neurology outpatient clinics in London and Kent, UK.

Participants Our aim was to recruit 50 TBI patients over 18 months. Follow up participants at 12 weeks using a battery of assessment scales to measure changes in aggressive behaviour (MOAS-primary outcome, IRQ) as well as global functioning (GOS-E, CGI) and quality of life (EQ-5D-5L, SF-12), and medication adverse effects (UKU).

Results Fourteen of 43 (33%) eligible patients were recruited, which is 28% of our original target of 50 participants. Six participants were randomised to the active arm of the trial and eight to the placebo arm over a 10-month period. Twelve out of 14 (86%) patients completed a follow up assessment at 12 weeks. At 12 weeks follow up, the scores of all outcome measures (MOAS, IRQ, CGI, EQ-5D-5L, SF-12) improved in both the placebo and the risperidone group. However, the placebo group did better than the risperidone

For peer review only

group. No severe adverse events were reported. The overall rate of adverse events remained low although was non-significantly higher in the risperidone group compared with the placebo group.

Conclusions Recruitment of adequate number of participants would be difficult in a future definitive RCT. Certain measures may increase the chance of recruitment. Feedback during process evaluation showed that once in the study, the participants did not find the trial procedure onerous. As placebo showed a better efficacy than risperidone in the current study, it is uncertain whether an adequately powered study will show any superiority of risperidone in future.

Trial registration number: ISRCTN30191436; 19th December 2016. **EudraCT no.** 2015-000641-23.

Strengths and limitations of this study

- We used a stringent RCT methodology for a feasibility trial.
- Once recruited in the study, the participants felt supported.
- A process evaluation helped to get positive and negative feedbacks from the participants.
- Recruitment has remained a major problem.
- Many regulatory authority delays hampered patient recruitment.

Introduction

Traumatic brain injury (TBI) is a major public health concern in the UK. Each year 1.4 million people attend hospitals in England and Wales with a recent TBI of whom around 200,000 people are admitted to hospital.[1,2] More and more patients with TBI are surviving longer now because of recent medical advances. However, it has been shown that long term consequences are common after TBI. Thornhill and colleagues (2000)[3] showed that, of patients admitted to hospital, 86% of those who sustained a severe TBI, 62% of those who sustained a moderate TBI and 55% of those who sustained a mild TBI had physical and emotional problems one year after the incident. In the first ever comprehensive assessment of neuropsychiatric consequences after TBI we found 32% of 18-65 years old patients (compared with 16% in the general population) followed up one year after TBI following discharge from a hospital had suffered from a psychiatric disorder.[4-7]

We found that 62% of TBI patients after one year of TBI showed more than one neurobehavioural symptom, and 40% showed three or more symptoms.[5] Of all the

For peer review only - http://bmjopen.bmj.com/site/about/guidelines.xhtml

For peer review only

neurobehavioural problems following TBI, aggression remains the most challenging. The long-term prevalence of aggressive behaviour following mild to severe TBI varies between 37% and 71% according to different studies.[8,9] Aggression affects quality of life of both the patients and their family carers and remains the most difficult problem to manage clinically. Both pharmacological and non-pharmacological interventions are used to manage this difficult behaviour. However, scientific evidence to suggest that any intervention works is lacking. As a result, front-line clinicians working with patients with these problems use a range of medications according to their clinical experience. This was evident from a recent audit we have carried out in 10 UK centres on 100 consecutive TBI patients who were treated for their aggressive behaviour with medication which showed that no less than 21 different medications had been used (unpublished data).

Two systematic reviews have confirmed the lack of high-quality clinical trials in this area.[10,11] While very small-scale RCTs (involving only 21, 11 and 4 participants respectively) of high doses of propranolol have been conducted, concerns about adverse events of these drugs at a high dose means they are not widely used in clinical practice. The most commonly used group of drugs in TBI are antipsychotics and mood stabilisers/ antiepileptic medications.[12] Although used widely in TBI, a systematic review did not find any good quality RCT based evidence without bias in support of mood stabilisers for the treatment of aggression in any patient group.[13] Among the antipsychotics the new generation of drugs such as risperidone, quetiapine, olanzapine and aripiprazole are used more often than the old generation of antipsychotics. However, because of lack of evidence and in the absence of any national/ international guidelines, the clinicians tend to choose antipsychotics depending on their, often limited, personal experience and specific clinical scenarios.

As risperidone is widely used in people with TBI for the treatment of aggression and is the only drug for which some evidence exists for efficacy in the management of aggression in any patient group such as ASD,[14] ID,[15] dementia[16] or schizophrenia[17] and as no such RCTs exist involving people with TBI,[10] we have chosen low dose risperidone as the active intervention drug for our placebo-controlled feasibility double-blind RCT for people with TBI. We have decided to carry out a feasibility RCT first as no large scale RCT of pharmacological intervention currently exists for the treatment of aggression in TBI patients. The aim of this feasibility study was to provide useful information to inform the design and execution of a future definitive RCT.

Aims and objectives

1) Improve the management of aggression among people with TBI by providing evidence for a pharmacological intervention.

For peer review only - http://bmjopen.bmj.com/site/about/guidelines.xhtml

2) Conduct a multi-centre parallel design placebo controlled (1:1 ratio) double-blind feasibility randomised controlled trial (RCT) of risperidone for the treatment of aggression in adults with TBI to assess feasibility for a substantive full scale definitive RCT in the future.
3) Recruit and randomise over 18 months 50 adults with TBI who are referred to clinicians primarily for the treatment of aggression from four centres within the greater London area to assess the potential rate of recruitment for a future definitive RCT.
4) To examine the views of trial participants about factors that could help ensure the success of a future definitive trial.

Methods

We conducted a multi-centre parallel design placebo controlled (1:1 ratio) double-blind feasibility RCT with embedded process evaluation.

Patient and Public involvement

The study was developed jointly with a group of 18 adults with TBI from a Headway centre in London. They advised us to be mindful of adverse effects of risperidone preferred a smaller dose for the RCT. We have used a smaller dose of risperidone in the trial and included a standard adverse event assessment scale. As suggested by the patient group, we have incorporated within the design a through examination by the clinicians of potential physical, psychological and social/environmental factors that may lead to aggression before considering the use of medication.

A patient with TBI attended all Trial Management Group meetings as a co-applicant. We also received feedback from a group of TBI patients through a Clinical Psychologist who acted as a facilitator. We have also received at the end of the study feedback through a process evaluation involving qualitative interviews of patients and their carers who took part in the trial.

Recruitment

Our aim was to recruit from four centres, namely the Imperial College NHS Healthcare Trust, the South West London and St. George's NHS Trust, the St. George's University Hospital Trust, and the Kent and Medway NHS Partnership Trust. Local clinicians or a nurse explained the study and handed over an outline leaflet describing the study and providing the researcher's contact details to the potential participants and their carers where applicable. Those patients who showed an interest in taking part in the study were contacted

For peer review only - http://bmjopen.bmj.com/site/about/guidelines.xhtml

by the researcher who described the study in more detail, gave them a detailed Research Ethics Committee approved information sheet and asked them to complete a consent form. After this the researcher completed a baseline pre-randomisation outcome assessment. A local clinician completed the Clinical Global Improvement-Severity (CGI-S) scale[18] and UKU adverse effect scale[19] and arranged for appropriate physical examination and blood tests.

Sample size

As this was a feasibility study, we did not carry out any formal power calculation but estimated that 40-50 is a reasonable number for a feasibility study which would allow us to carry out a sample size calculation for the full future proposed RCT.[20] A general guide for number of participants necessary for a feasibility study is 30 patients or more.[21]

Inclusion/ exclusion criteria

Inclusion criteria:

a) 18 years or older
b) patients with a confirmed clinical diagnosis of TBI using the Mayo Clinic criteria,[22] which happened at least six months prior to recruitment
c) referred to the clinicians for the management of aggression and for whom the clinician is considering a pharmacological intervention for this problem after investigating and addressing physical, psychological and social triggers.

Exclusion criteria:

a) patient suffering from Post-Traumatic Amnesia (PTA)
b) co-morbid serious mental illness such as schizophrenia and other psychoses, bipolar disorder, major depressive disorder, personality disorder, and dementia
c) patients who were already on an antipsychotic drug or any other drug that may interact with risperidone at the time of randomisation
d) any other contraindication for using risperidone including a previous history of severe adverse events
e) patients with no fixed abode or any other reason for which compliance with study medication and monitoring could pose a major problem
f) A history of definite neurogenic seizures within the last three months.

Active drug dose

For peer review only - http://bmjopen.bmj.com/site/about/guidelines.xhtml

We used a flexible dose of risperidone. We started with 1 mg once daily dose and increased the dose if necessary, to 1 mg twice daily on week 2, 2 mgs at night and 1 mg in the morning on week 3, and 2 mgs twice daily from week 4 onwards for the rest of the 12 weeks study period. Equivalent number of placebo capsules were also administered to the appropriate participants. The local clinicians who were blind to the allocation decided whether a dose increase, or decrease is warranted depending on major clinical improvement or troublesome adverse events. Local clinicians were allowed to use PRN medication if necessary.

Outcome measures

Feasibility outcome measures

Our criteria for demonstrating success of the feasibility study were: (a) recruitment of at least 80% of the sample who met inclusion criteria after screening and (b) completion of follow up interviews at 12 weeks by at least 75% of the study participants.

Clinical outcome measures

Primary outcome measure:

We used Modified Overt Aggression Scale (MOAS) to assess improvement in aggression. The MOAS is a simple and widely used 4-item scale that measures verbal aggression along with physical aggression towards other people, property and self. The MOAS is a valid and reliable instrument which has been used in many pharmacological intervention studies of aggression in different neuropsychiatric patient groups including TBI.[23]

Secondary outcome measures:

1) Glasgow Outcome Scale-Extended version (GOS-E),[24] which is a widely used global outcome measure of social functioning following TBI,
2) Irritability Questionnaire (IRQ),[25] which has both a patient and a carer version,
3) Hospital Anxiety and Depression Scale (HADS),[26]
4) Clinical Global Impression-Improvement (CGI-I) and CGI-S (CGI-Severity) Scales,[27]
5) Udvalg for Kliniske Undersøgelser (UKU) scale[28] to assess adverse effects of risperidone, and
6) Two widely used quality of life (QoL) measures, namely EQ-5D-5L[29] and SF-12.[30]

All these measures were used at baseline and at 12 weeks follow up.

For peer review only - http://bmjopen.bmj.com/site/about/guidelines.xhtml

For peer review only

We used a proforma specifically designed for this study to collect demographic data such as age, gender but also other relevant information such as cause and severity of TBI etc.

Researcher completed MOAS based on information from both the patients and the carers (where available).

Concealment and randomisation

Remote web-based randomisation was undertaken through a fully automated service operated by an external organisation 'Sealed Envelope'. Our aim was to randomise an equal number of participants to risperidone and placebo. We used random permuted blocks stratified by study centres (n=4). Sealed Envelope designed the randomisation list. The central pharmacy at Royal Free Hospital put the unique randomisation number on each bottle of active medication and placebo according to the list provided by Sealed Envelope. There was a 24 hours telephone number available for the clinicians and research team if the randomisation code needed to be broken.

Statistical analyses of clinical data

Primary efficacy analysis

The primary outcome was the patient reported MOAS score, measured at 12 weeks. As this is a feasibility study, no formal hypothesis tests were performed to statistically compare the two study groups. Instead the following analysis was performed:

1) Descriptive statistics for the outcome at 12 weeks in each study group, and also for the change in outcome from baseline to 12 weeks.
2) The mean difference in outcome between study arms (or median if deemed more appropriate).
3) The standard deviation of the MOAS score.
4) The size of the correlation between MOAS score at baseline and 12 weeks using Spearman's rank correlation, which was preferred to the Pearson method due to the distribution of the scores.

Secondary efficacy analyses

All secondary outcomes were analysed descriptively, with no formal hypothesis tests performed. Summary statistics for the outcomes at Baseline and Week 12 were calculated for each of the two study groups.

For peer review only - http://bmjopen.bmj.com/site/about/guidelines.xhtml

For outcomes regarded as being measured on a continuous scale, summary statistics were also produced for the changes from Baseline to Week 12. Changes were not considered appropriate for ordinal or categorical outcomes.

Safety analyses

The main safety outcome was the occurrence of adverse events (AEs) and serious adverse events (SAEs). The total number of adverse events were reported. Additionally, a summary of all adverse events was made, with following summaries being made:

1) Description of adverse events.
2) Seriousness (serious, not serious).
3) Expectedness (expected, unexpected).
4) Causality due to study participation (definite, probable, possible, unlikely, unrelated).

Process evaluation

A process evaluation using a qualitative method examined trial recruitment and the feasibility and acceptability of trial procedures from the perspective of patients and their carers. We planned to undertake semi-structured qualitative interviews with trial participants, and their carers, purposively sampled to represent site and treatment allocation.

Interviews were conducted using an interview topic. This was developed initially by the project team but modified as the feasibility study progressed in response to emergent themes. These interviews were audio-recorded, transcribed and analysed using a thematic framework approach[31] and managed using NVivo[32] computer software. An initial framework was based on the study aims of how feasible it was to undertake a trial. This was further developed using analytic induction from early interviews and iteratively revised as data collection and analysis progressed.

The study received a favourable ethical opinion from the London-Westminster Research Ethics Committee on behalf of the NHS Health Research Authority.

Results

Fourteen of the 43 (33%) eligible patients were recruited between March 2017 and January 2018 (see Figure 1). Eight patients received placebo and six risperidone (see Figure 1). The demographic characteristics of study participants are summarised in Table 1. The mean age of patients was 43 and 39 in the placebo and risperidone groups respectively. Over 60% of both groups were male.

For peer review only - http://bmjopen.bmj.com/site/about/guidelines.xhtml

Feasibility outcome data

Three patients started but did not complete the study (attrition rate 21%) (two because of the adverse effects and one was unable to continue because of work commitments). One of these three patients took part in the follow up assessment. Overall follow up assessment was available on 12 patients (follow up rate 86%).

Original recruitment target was 50 patients from four centres over 18 months (0.69 patient/ centre/ month). One centre did not recruit any patient and another centre recruited only one patient who did not complete the trial. The overall recruitment rate was 0.3 patient/ centre/ month across all four centres (21% of the target rate) over 10 months period. One of the four centres is a University centre (Imperial College) led by a Professor of Neurology (DS), which is already involved in several research studies involving patients with a head injury. The recruitment rate from the research active (University) centre was 0.78/ month (113% of the target rate). The recruitment rate between two active recruiting centres (the University centre and the Kent centre where a consultant neuropsychiatrist (MB), with interest in research was the local Principal Investigator) was 93% of the target rate. The research active centre held a patient database which helped the researcher to screen for potential recruits. This meant not having to go through each individual patient's case notes separately who were in the local clinician's caseload or even having to screen patients prospectively.

Quantitative outcome data

The data suggest slightly higher MOAS values at baseline in the Placebo group. At 12 weeks, MOAS values had reduced in both groups, down to a similar level in the two groups. Due to the higher baseline values, the reduction in scores from baseline to 12 weeks was greater in the Placebo group than in the Risperidone group (see Table 2). In order to aid a possible future sample size calculation (using an ANCOVA approach), the association between MOAS total score at baseline and 12 weeks was assessed. Spearman's rank correlation gave a correlation of 0.04 between timepoints. This suggests little relationship between the scores at the two timepoints. MOAS subdomain data are presented in Table 3.

The patient reported IRQ measures (severity and frequency) were similar in the two groups at baseline. Both components showed slightly lower values in the Risperidone group at Week 12, with a slightly greater reduction in both scores in this group. Both carer measures were slightly higher at baseline in the Risperidone group. Slightly larger reductions in scores were observed in this group, giving similar severity and frequency scores in both groups at Week 12 (see Table 4).

For peer review only - http://bmjopen.bmj.com/site/about/guidelines.xhtml

The clinician reported CGI results suggested a slight imbalance at Baseline. 80% of Risperidone patients were mildly ill or less at this timepoint, compared with only 29% of the Placebo group. At Week 12 all patients in the Risperidone group were mildly ill or less, compared with less than 60% of the Placebo group. The clinician reported improvements in CGI scores suggested that 40% of patients were improved in the Risperidone group, compared with just less than 60% in the Placebo group. The slightly higher improvement rate in the Placebo group could be attributable to the worse scores at Baseline in this group, leaving more room for improvement (see Table 5). The same picture was revealed for the two quality of life (QoL) scales scores, namely SF-12, and EQ-5D-5L, GOS-E, and HADS score.

No severe adverse events were reported. The overall rate of adverse events remained low although it was non-significantly higher in the Risperidone group compared with the Placebo group (see Table 6).

Qualitative outcome data

Eight patients and two of their carers were interviewed and the data were analysed by the researcher (LA), under the supervision from the qualitative investigator (TW). The analysis focused on the factors that had a positive influence by facilitating or promoting participation in the study and, alternatively, those factors that exerted a negative influence hindering participation.

Key factors exerting a positive influence on participation reported by patients and their carers included a belief in the positive benefit of research. This may have been expressed as (a) a perception that the trial may help achieve their 'desire to get better', or (b) satisfy an altruistic motivation to participate in research that might help other people in a similar situation. Some also reported (c) previous positive experiences in taking part in research. Also, significant to maintaining engagement were (d) the very straightforward and easy research procedures, and (e) the high level of trust and support engendered by weekly telephone contact from the researcher (LA) and (f) good relations with the referring doctor. Participation was also associated with (g) a higher educational level of the participants.

The analysis showed that the key reasons for patients not taking part in the study were; (a) a reluctance to change treatment, (b) perceived adverse effects of the study medication, (c) lack of understanding of the reason why the study is undertaken as this was not clearly explained to them, (d) lack of insight into their own condition, so the potential participants did not see their behaviour as a problem needing any intervention (e) unwillingness to engage with treatment regime or trial procedures and (f) objection to randomisation manifested as a reluctance particularly on the part of the carers regarding

For peer review only - http://bmjopen.bmj.com/site/about/guidelines.xhtml

potential allocation into the placebo arm which they thought might make the patient's behaviour worse and difficult to manage.

Furthermore, many participants, particularly those who had memory and attention problem, found it tiring to complete so many assessment measures some of which were quite lengthy. Similarly, memory problems may have affected the consent process thus making some potential patients ineligible for recruitment.

Discussion

In the current study risperidone did not show a superiority over placebo at follow up. If anything, placebo group showed a better outcome over risperidone. Therefore, it is difficult to know whether an adequately powered RCT in future will show superiority of risperidone over placebo, which could only be confirmed by carrying out a definitive RCT in future.

As the purpose of the current study is to assess feasibility of a future definitive study, we concluded that if recruitment happens at the same overall rate of the current study, then a definitive RCT would be difficult to conduct in the future. Our rate of recruitment fell short of our original goal because two of the four centres failed to recruit participants but the follow up was within the expected range. Therefore, if recruitment happens at the same rate as the two active recruiting centres (described in the results section) in the current study, a future RCT would be feasible.

An approximate sample size calculation done by the study statistician (PB) shows that for a 5% significance level and 90% power, 72 patients per group and 144 in total will be required. Allowing for a 20% attrition rate, a total of 180 patients need to be recruited. If we have the same rate of recruitment (0.3 patient/ centre/ month) in a future adequately powered RCT, we will need to recruit from 17 centres over a 3-year period to achieve a recruitment rate of 180 patients. However, if we can recruit at the same rate as the 2 active recruiting centres in our study (0.64 patient/ centre/ month), we will need to recruit from 15.6 centres over 18 months to achieve the recruitment of 180 patients.

We also concluded that an adequate number of participants could not be recruited in a future definitive full RCT from Neuropsychiatry clinics alone. We believe that to recruit an adequate number of participants in the future, it would be necessary to broaden the range of services to include Neurorehabilitation centres and possibly Trauma centres. Approaching voluntary organisations may be fruitful as many of them run Day centres for TBI patients but the experience of involving Headways in London in the current study is disappointing. It is not clear whether approaching primary care would be effective. Screening the primary care patient list for potential participants would be difficult and a large number of primary care

For peer review only - http://bmjopen.bmj.com/site/about/guidelines.xhtml

sites would be required to recruit an adequate number of participants, which may not be feasible given the resource available for research.

Our experience shows that research active centres are likely to recruit an adequate number of participants in a future RCT as they already have the infra-structure and the patients attending these centres are already familiar with research procedures and may have already taken part in other research studies. However, the downside may be that patients may have already been recruited in one or more studies, therefore, would be difficult to recruit into yet another study.

We found that having a database of patients with clinical information is useful as some Principal Investigators (PIs) found it difficult and time consuming to having to go through a large number of patients' clinical records in order to identify suitable patients for the study. Similarly, having had an enthusiastic junior/ trainee doctor or a specialist/ senior clinic nurse to help may be very useful in both recruitment and the running of the study. We felt that the local CSO involvement may or may not be helpful, which is dependent on the quality and quantity of the local resource. However, a local PI with some academic interest is likely to help with the recruitment.

We found the way the project is presented to the potential participant is of utmost importance. Therefore, we feel that a face to face meeting with the potential patients explaining the study properly than a telephone call is more likely to increase the chance of recruitment. Regular support such as weekly telephone calls (as was the case in our study) is likely to retain the patient in the study. We feel that the offer of some non-pharmacological intervention such as 'anger management' along with either placebo or an active drug is likely to increase the recruitment rate. However, this will also involve the additional NHS excess treatment cost and local resource which may not be available.

We found that it is important to carefully choose exclusion criteria as in our study initial criterion of excluding patients with a history of epilepsy meant exclusion of some otherwise eligible patients. We later revised the exclusion criterion to 'history of a definite neurogenic seizure within the last three months.' Feedback from participants and the patient advisory group emphasised the importance of writing the patient information sheet in an accessible format as many patients have problem with attention and memory. It is also important to consider carefully the number of outcome measures need to be used and their length as several patients in our study found completing so many scales tiring, particularly if they had memory problem and suffered from tiredness, impaired attention and apathy.

Therefore, a future definitive RCT will have to take these issues into account and design the study accordingly to mitigate against the factors mentioned above in order to be successful in recruiting an adequate number of participants and keep the attrition rate to a

For peer review only - http://bmjopen.bmj.com/site/about/guidelines.xhtml

minimum. It is also necessary to have a more developed infrastructure that will allow recruitment of TBI patients in the chronic phase. Part of the problem is that well set up chronic TBI clinics are not common in the UK which is not helpful for recruitment into such RCTs. It is equally important to have some degree of research capability aligned to the clinics that will allow this type of research to be undertaken. Therefore, for a successful RCT we need a) TBI Follow-up clinics to be in place, b) a database for these clinics, c) a senior clinician engaged enough to facilitate the study and d) a junior person who is enthusiastic and prepared to help with the study. We think if these are in place then recruitment would be possible as we have shown in our study.

For peer review only

Authors' contribution

SD was involved in the conception and design of the study, supervised data collection, and wrote the funding application, the study protocol and this manuscript. LA contributed to study protocol, collected all research data and liaised with recruiting sites, carried out qualitative interviews with patients and their carers, and contributed to this manuscript. VL contributed to study protocol, overseen regulatory procedures including Ethics and MHRA approval and supervised the day to day running of the trial, and contributed to this manuscript. MB contributed to the study protocol, recruited patients and contributed to this manuscript. LL recruited patients and contributed to this manuscript. TW contributed to the study protocol, supervised qualitative interview data analysis, and contributed to this manuscript. DS contributed to study protocol, recruited patients and contributed to this manuscript. PB helped with the study design, developed a statistical analysis plan (SAP) and carried out the statistical analyses, and contributed to this manuscript. MC contributed to the study protocol, supervised day to day dealings with the Sponsor and contributed to this manuscript.

Competing interest

None declared.

Funding

This article presents independent research funded by the National Institute for Health Research (NIHR), UK under its Research for Patient Benefit (RfPB) Programme (Grant Reference Number PB-PG-1013-32054). The views expressed are those of the authors and not necessarily those of the NHS, the NIHR or the Department of Health, UK.

For peer review only - http://bmjopen.bmj.com/site/about/guidelines.xhtml

Availability of supporting data

Only the designated trial investigators have access to the personal data of the participants and to the final dataset. The original e-CRF pages generated during the study is the property of the sponsor (Central and North West London Partnership NHS Foundation Trust, UK). Regulatory bodies if appropriate may request access to personal data. There may be opportunity to share data anonymously if necessary and appropriate after proper authorisation and approval are obtained.

Ethical approval and consent to participate

A favourable ethical opinion and consent to participate were granted by the London and Westminster Research Ethics Committee, NHS-Health Research Authority, Research Ethics Service, UK on the 30th December 2015. All participants have provided a written consent before participating in the study.

Access to source data/documents

The investigator(s)/ institution(s) will permit trial-related monitoring, audits, REC review, and regulatory inspection(s), providing direct access to source data/documents. Trial participants were informed of this during the informed consent discussion. Participants consented to provide access to their medical records.

Ethics and regulatory requirements

The sponsor has ensured that the trial Protocol, study Information Sheets, Consent Forms, GP letter and submitted supporting documents have been approved by the MHRA and a main REC, prior to any patient recruitment. The protocol and all agreed substantial protocol amendments, were documented and submitted for ethical and, other necessary, regulatory approval prior to implementation according to Sponsor Specific Operational Procedures (SOPs).

Acknowledgement

The Imperial Biomedical Research Centre Facility which is funded by the National Institute of Health Research, UK provided support for the study.

Supplementary file: Protocol.

For peer review only - http://bmjopen.bmj.com/site/about/guidelines.xhtml

For peer review only

For peer review only - http://bmjopen.bmj.com/site/about/guidelines.xhtml

References

1. Deb S. ICD-10 codes detect only a proportion of all head injury admissions. *Brain Injury* 1999;13(5):369-73.

2. National Institute for Health and Care Excellence (NICE). Triage, assessment, investigation and early management of head injury in children, young people and adults. Partial update of NICE CG56 (Draft for consultation). August 2013, NICE, London UK (nice.org.uk).

3. Thornhill S, Teasdale GM, Murray GD, McEwan J, Roy CW, Penny KI. Disability in young people and adults one year after head injury: prospective cohort study. *British Medical Journal* 2000;320:1631-5.

4. Deb S, Lyons I, Koutzoukis C. Neuropsychiatric sequelae one year after a minor head injury. *Journal of Neurology Neurosurgery and Psychiatry* 1998;65:899-902.

5. Deb S, Lyons I, Koutzoukis C. Neurobehavioural symptoms one year after a head injury. *British Journal of Psychiatry* 1999;174:360-5.

6. Deb S, Burns J. Neuropsychiatric consequences of traumatic brain injury: a comparison between two age groups. *Brain Injury* 2007;21(3):301-7.

7. Deb S, Lyons I, Koutzoukis C, Ali I, McCarthy G. Rate of psychiatric illness one year after traumatic brain injury. *American Journal of Psychiatry* 1999;156:374-8.

8. Silver JM, Yudofsky SC, Anderson KE. Aggressive behavior, in Textbook of Traumatic Brain Injury. Edited by Silver JM, McAllister TW, Yudofsky SC. Washington, DC, *American Psychiatric Association Publishing* 2011; pp. 225–38.

9. Kim E, Lauterbach EC, Reeve A, Arciniegas DB, Coburn KL, Mendez MF, Rummans TA, Coffey EC. Neuropsychiatric Complications of Traumatic Brain Injury: A Critical Review of the Literature (A Report by the ANPA Committee on Research). *Journal of Neuropsychiatry and Clinical Neurosciences* 2007;19:106-27.

10. Sadavoy J, Lanctôt KL, Deb S. Management of behavioural and psychological symptoms of dementia and acquired brain injury. In 'Cambridge Textbook of Effective Treatments in Psychiatry' (Eds. P. Tyrer & K. R. Silk), *Cambridge University Press* 2008, Cambridge. Pp. 187-216.

11. Fleminger S, Greenwood RRJ, Oliver DL. Pharmacological management for agitation and aggression in people with acquired brain injury (review) 2006. *The Cochrane Library*, Issue 4 (www.thecochranelibrary.com).

For peer review only - http://bmjopen.bmj.com/site/about/guidelines.xhtml

12. Deb S, Crownshaw T. The role of pharmacotherapy in the management of behaviour disorders in traumatic brain injury patients. *Brain Injury* 2004;18(1):1-31.

13. Huband N, Ferriter M, Nathan R, Jones H. Antiepileptics for aggression and associated impulsivity. *Cochrane Database of Systematic Reviews* 2010; Issue 2: Art. No. CD003499. Doi: 10.1002/14651858.CD003499.pub3.

14. Jones RM, Arlidge J, Gillham R, Reagu S et al. Efficacy of mood stabilisers in the treatment of impulsive or repetitive aggression: systematic review and meta-analysis. *British Journal of Psychiatry* 2011;198:93-8.

15. Unwin GL, Deb S. Efficacy of atypical antipsychotic medication in the management of behaviour problems in children with intellectual disabilities and borderline intelligence: a systematic review. *Research in Developmental Disabilities* 2011;32:2121-33.

16. Ballard CG, Waite J, Birks J. Atypical antipsychotics for aggression and psychosis in Alzheimer's disease (Review). *Cochrane Database of Systematic Reviews* 2006; Issue 1. Article no. CD003476. Doi: 10.1002/14651858.CD003476.pub2.

17. Marder SR, Davis JM, Chouinard G. The effects of risperidone on the five dimensions of schizophrenia derived by factor analysis: combined results of the North American Trials. *J Clin Psychiatry* 1997;58:538-46.

18. Guy W. Early Clinical Drug Evaluation Unit (ECDEU) assessment manual for psychopharmacology: Revised. Bethesda MD 1976; *National Institute of Mental Health*.

19. Lingjaerde O, Ahlfos UG, Bech P, Dencker SJ. Elgen K. The UKU side effect rating scale: a new comprehensive rating scale for psychotropic drugs, and a cross-sectional study of side effects in neuroleptic-treated patients. *Acta Psychiatrica Scandinavica* 1987;334 (Suppl.):1-100.

20. Lancaster GA, Dodd S, Williamson PR. Design and analysis of pilot studies: recommendations for good practice. *Journal of Evaluation in Clinical Practice* 2002;10:307-12.

21. Browne RH. On the use of a pilot sample for size determination. *Statistics & Medicine* 1995;14:1933-40.

For peer review only - http://bmjopen.bmj.com/site/about/guidelines.xhtml

22. Malec JF, Brown AW, Leibson CL, Flaada JT, Manderkar JN, Diehl NN, Perkins PK. The Mayo classification system for traumatic brain injury severity. *Journal of Neurotrauma* 2007;24:1417-24.

23. Ratey JJ, Gutheil CM. The measurement of aggressive behaviour: reflections on the use of the Overt Aggression Scale and the Modified Overt Aggression Scale. *Journal of Neuropsychiatry* 1991;3:557-60.

24. Wilson JTL, Pettigrew LEL P, Teasdale GM. Structured interviews for the Glasgow Outcome Scale and the Extended Glasgow Outcome Scale: Guidelines for their use. *Journal of Neurotrauma* 1998;15(8):573-85.

25. Craig KJ, Heitanen H, Markova IS, Berrios GE. The Irritability Questionnaire: a new scale for the measurement of irritability. *Psychiatry Research* 2008;159:367-75.

26. Zigmond AS, Snaith RP. The Hospital Anxiety and Depression Scale. *Acta Psychiatrica Scandinavica* 1983;67:361-70.

27. National Institute of Mental Health. CGI Clinical Global Impression Scale-NIMH, *Psychopharmacology Bulletin* 1985;21:839-44.

28. Lingjaerde O, Ahlfos UG, Bech P, Dencker SJ. Elgen K. The UKU side effect rating scale: a new comprehensive rating scale for psychotropic drugs, and a cross-sectional study of side effects in neuroleptic-treated patients. *Acta Psychiatrica Scandinavica* 1987;334(Suppl.):1-100.

29. Herdman M, Gudex C, Lloyd A, Janssen MF, Kind P, Parkin D, Bonsel G, Badia X. Development and preliminary testing of the new five-level version of EQ-5D (EQ-5D-5L). *Quality of Life Research* 2013;20(10):1727-36.

30. Ware JE. JR., Mark K, Susan DK. A 12-Item Short-Form Health Survey: Construction of Scales and Preliminary Tests of Reliability and Validity. *Medical Care* 1996;34(3):220-33.

31. Hesse-Biber S, Leavy P. The Practice of Qualitative research. *Thousand Oaks*, CA 2006; Sage Publications.

32. NVivo 12 Plus. QSR International UK Ltd., 2019; London, UK.

Table 1: Patient demographics

Variable	Category	Placebo	Risperidone

For peer review only - http://bmjopen.bmj.com/site/about/guidelines.xhtml

Number patients	-	8	6
Gender	Female	3 (38%)	1 (17%)
	Male	5 (62%)	5 (83%)
Age	-	43.1 ± 11.3 years	39.3 ± 8.7 years
TBI Cause	Assault	2 (25%)	0 (0%)
	Fall	3 (38%)	1 (17%)
	Road Traffic Accident	3 (38%)	3 (50%)
	Other	0 (0%)	2 (33%)
Initial severity (*)	Mild	2 (33%)	1 (17%)
	Moderate	1 (17%)	3 (50%)
	Severe	3 (50%)	2 (33%)
History of coma	No	5 (62%)	3 (50%)
	Yes	3 (38%)	3 (50%)
History of amnesia	No	5 (62%)	2 (33%)
	Yes	3 (38%)	4 (67%)

Summary statistics are: Mean ± SD or Number (percentage)

(*) Missing data for 2 patients in Placebo group

Table 2: Summaries of patient reported MOAS

Timepoint	Group	N	Mean ± SD	Median [IQR]	Range
Baseline (+)	Placebo	7	11.6 ± 9.6	8 [2, 24]	2, 25
	Risperidone	5	5.0 ± 3.0	6 [2, 8]	2, 9
Week 12	Placebo	7	2.0 ± 2.4	1 [0, 3]	0, 7
	Risperidone	5	2.2 ± 3.9	0 [0, 6]	0, 9

For peer review only - http://bmjopen.bmj.com/site/about/guidelines.xhtml

Change (*)	Placebo<br>Risperidone	7<br>5	-9.5 ± 10.5<br>-2.8 ± 4.8	-4 [-23, -1]<br>-2 [-8, 2]	-25, -1<br>-9, 3

(+) Summary statistics only for patients with values at 12 weeks

(*) Calculated as value at Week 12 minus value at baseline

Table 3: MOAS subdomain scores

		Placebo		Risperidone	
Outcome	Timepoint	N	Summary	N	Summary
MOAS patient - Verbal	Baseline (+)	7	4.7 ± 2.4	5	3.4 ± 1.7
	Week 12	7	1.4 ± 1.3	5	1.0 ± 1.4
	Change (*)	7	-3.3 ± 2.7	5	-2.4 ± 2.3
MOAS patient - Against property	Baseline (+)	7	2.0 ± 2.1	5	0.2 ± 0.4
	Week 12	7	0.3 ± 0.8	5	0.2 ± 0.4
	Change (*)	7	-1.7 ± 2.4	5	0.0 ± 0.0
MOAS patient - Autoaggression	Baseline (+)	7	0.6 ± 0.8	5	0.4 ± 0.9
	Week 12	7	0.0 ± 0.0	5	0.0 ± 0.0
	Change (*)	7	-0.6 ± 0.8	5	-0.4 ± 0.9
MOAS patient - Against people	Baseline (+)	7	0.3 ± 0.8	5	0.0 ± 0.0
	Week 12	7	0.0 ± 0.0	5	0.2 ± 0.4
	Change (*)	7	-0.3 ± 0.8	5	0.2 ± 0.4
MOAS carer - Total score	Baseline (+)	5	8.4 ± 8.4	3	10.3 ± 6.5
	Week 12	5	2.8 ± 3.1	3	3.7 ± 3.8
	Change (*)	5	-5.6 ± 5.6	3	-6.7 ± 3.2

For peer review only

(+) Summary statistics only for patients with values at 12 weeks

(*) Calculated as value at Week 12 minus value at baseline

Table 4: Irritability Questionnaire (IRQ) scores in two groups

Outcome	Timepoint	Placebo N	Placebo Mean ± SD	Risperidone N	Risperidone Mean ± SD
IRQ patient - Severity	Baseline (+)	7	34 ± 6	5	37 ± 19
	Week 12	7	26 ± 7	5	25 ± 14
	Change (*)	7	-8 ± 9	5	-13 ± 13
IRQ patient - Frequency	Baseline (+)	7	40 ± 7	5	41 ± 10
	Week 12	7	29 ± 6	5	27 ± 15
	Change (*)	7	-11 ± 7	5	-14 ± 18
IRQ carer - Severity	Baseline (+)	5	10 ± 4	5	14 ± 5
	Week 12	5	7 ± 4	5	7 ± 4
	Change (*)	3	-3 ± 2	5	-6 ± 2
IRQ carer - Frequency	Baseline (+)	5	10 ± 4	3	15 ± 8
	Week 12	5	9 ± 6	3	10 ± 8
	Change (*)	5	-1 ± 5	3	-5 ± 2

(+) Summary statistics only for patients with values at 12 weeks

(*) Calculated as value at Week 12 minus value at baseline

Table 5: Clinician reported Clinical Global Impression (CGI)

Outcome	Timepoint	Category	Placebo N (%)	Risperidone N (%)
CGI clinician - Severity	Baseline (+)	Normal	0 (0%)	1 (20%)
		Borderline ill	2 (29%)	0 (0%)
		Mildly ill	0 (0%)	3 (60%)
		Moderately ill	4 (57%)	1 (20%)
		Markedly ill	1 (14%)	0 (0%)

For peer review only - http://bmjopen.bmj.com/site/about/guidelines.xhtml

		Severely ill	0 (0%)	0 (0%)
	Week 12	Normal	2 (29%)	2 (40%)
		Borderline ill	0 (0%)	1 (20%)
		Mildly ill	2 (29%)	2 (40%)
		Moderately ill	2 (29%)	0 (0%)
		Markedly ill	1 (14%)	0 (0%)
		Severely ill	0 (0%)	0 (0%)
CGI clinician - Improvement	Change	Very much improved	2 (29%)	1 (20%)
		Much improved	1 (14%)	1 (20%)
		Minimally improved	1 (14%)	0 (0%)
		No change	2 (29%)	3 (60%)
		Minimally worse	1 (14%)	0 (0%)
		Much worse	0 (0%)	0 (0%)

(+) Summary statistics only for patients with values at 12 weeks

Table 6: Adverse event summaries

Outcome	Category	Placebo	Risperidone
Total Adverse events (AE)	-	6	10
Patients with AEs	-	4 (50%)	5 (83%)
AEs per patient	-	0.8 ± 0.9 [0, 2]	1.7 ± 1.8 [0, 5]
Adverse events category	Anger	0 (0%)	2 (20%)
	Anxiety	0 (0%)	1 (10%)
	Chest pain	0 (0%)	1 (10%)
	Cough	1 (17%)	0 (0%)
	Fatigue	1 (17%)	5 (50%)
	Insomnia	0 (0%)	1 (10%)
	Loss appetite	1 (17%)	0 (0%)
	Slips, trips, falls	3 (50%)	0 (0%)

For peer review only

For peer review only - http://bmjopen.bmj.com/site/about/guidelines.xhtml

Seriousness	Not serious	6 (100%)	10 (100%)
	Serious AE	0 (0%)	0 (0%)
Severity	Mild	4 (67%)	7 (70%)
	Moderate	2 (33%)	2 (20%)
	Severe	0 (0%)	1 (10%)
Relationship to drug	Unrelated	2 (33%)	1 (10%)
	Unlikely	3 (50%)	2 (20%)
	Possibly	1 (17%)	3 (30%)
	Probably	0 (0%)	4 (40%)
	Definitely	0 (0%)	0 (0%)
Outcome	Resolved	6 (100%)	10 (100%)

Summary statistics are: Mean ± standard deviation [range], or number (percentage)

For peer review only

For peer review only - http://bmjopen.bmj.com/site/about/guidelines.xhtml

Figure 1: CONSORT diagram:

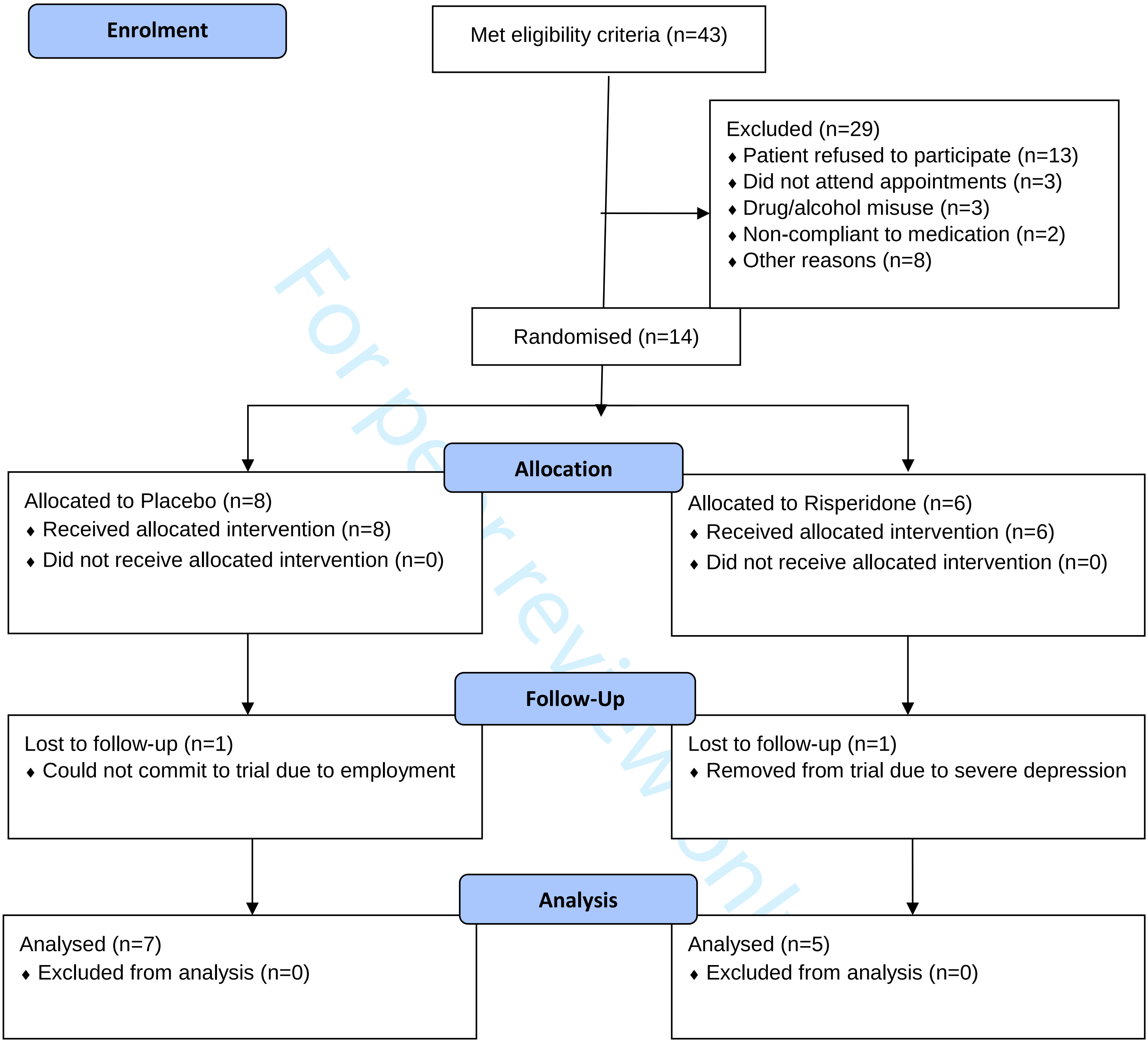

For peer review only

STANDARD PROTOCOL ITEMS: RECOMMENDATIONS FOR INTERVENTIONAL TRIALS

SPIRIT 2013 Checklist: Recommended items to address in a clinical trial protocol and related documents*

Section/item	Item No	Description	Addressed on page number
**Administrative information**			
Title	1	Descriptive title identifying the study design, population, interventions, and, if applicable, trial acronym	_____3________
Trial registration	2a	Trial identifier and registry name. If not yet registered, name of intended registry	______4_______
	2b	All items from the World Health Organization Trial Registration Data Set	______4_______
Protocol version	3	Date and version identifier	________4______
Funding	4	Sources and types of financial, material, and other support	________24______
Roles and responsibilities	5a	Names, affiliations, and roles of protocol contributors	______1,2______
	5b	Name and contact information for the trial sponsor	______23_______
	5c	Role of study sponsor and funders, if any, in study design; collection, management, analysis, and interpretation of data; writing of the report; and the decision to submit the report for publication, including whether they will have ultimate authority over any of these activities	______23_______
	5d	Composition, roles, and responsibilities of the coordinating centre, steering committee, endpoint adjudication committee, data management team, and other individuals or groups overseeing the trial, if applicable (see Item 21a for data monitoring committee)	______18_______

For peer review only - http://bmjopen.bmj.com/site/about/guidelines.xhtml

**Introduction**			
Background and rationale	6a	Description of research question and justification for undertaking the trial, including summary of relevant studies (published and unpublished) examining benefits and harms for each intervention	_______5________
	6b	Explanation for choice of comparators	_______6________
Objectives	7	Specific objectives or hypotheses	_______7________
Trial design	8	Description of trial design including type of trial (eg, parallel group, crossover, factorial, single group), allocation ratio, and framework (eg, superiority, equivalence, noninferiority, exploratory)	________7_______
**Methods: Participants, interventions, and outcomes**			
Study setting	9	Description of study settings (eg, community clinic, academic hospital) and list of countries where data will be collected. Reference to where list of study sites can be obtained	_______9________
Eligibility criteria	10	Inclusion and exclusion criteria for participants. If applicable, eligibility criteria for study centres and individuals who will perform the interventions (eg, surgeons, psychotherapists)	_____7,8______
Interventions	11a	Interventions for each group with sufficient detail to allow replication, including how and when they will be administered	_____10,11_______
	11b	Criteria for discontinuing or modifying allocated interventions for a given trial participant (eg, drug dose change in response to harms, participant request, or improving/worsening disease)	______10,11_____
	11c	Strategies to improve adherence to intervention protocols, and any procedures for monitoring adherence (eg, drug tablet return, laboratory tests)	______13,16_____
	11d	Relevant concomitant care and interventions that are permitted or prohibited during the trial	______11_________
Outcomes	12	Primary, secondary, and other outcomes, including the specific measurement variable (eg, systolic blood pressure), analysis metric (eg, change from baseline, final value, time to event), method of aggregation (eg, median, proportion), and time point for each outcome. Explanation of the clinical relevance of chosen efficacy and harm outcomes is strongly recommended	_____12,13_______
Participant timeline	13	Time schedule of enrolment, interventions (including any run-ins and washouts), assessments, and visits for participants. A schematic diagram is highly recommended (see Figure)	______10,11_____

For peer review only - http://bmjopen.bmj.com/site/about/guidelines.xhtml

Sample size	14	Estimated number of participants needed to achieve study objectives and how it was determined, including clinical and statistical assumptions supporting any sample size calculations	_____10________
Recruitment	15	Strategies for achieving adequate participant enrolment to reach target sample size	_____8,9_______
**Methods: Assignment of interventions (for controlled trials)**			
Allocation:			
Sequence generation	16a	Method of generating the allocation sequence (eg, computer-generated random numbers), and list of any factors for stratification. To reduce predictability of a random sequence, details of any planned restriction (eg, blocking) should be provided in a separate document that is unavailable to those who enrol participants or assign interventions	____14,15______
Allocation concealment mechanism	16b	Mechanism of implementing the allocation sequence (eg, central telephone; sequentially numbered, opaque, sealed envelopes), describing any steps to conceal the sequence until interventions are assigned	______15_______
Implementation	16c	Who will generate the allocation sequence, who will enrol participants, and who will assign participants to interventions	_____15________
Blinding (masking)	17a	Who will be blinded after assignment to interventions (eg, trial participants, care providers, outcome assessors, data analysts), and how	______15_______
	17b	If blinded, circumstances under which unblinding is permissible, and procedure for revealing a participant's allocated intervention during the trial	______15_______
**Methods: Data collection, management, and analysis**			
Data collection methods	18a	Plans for assessment and collection of outcome, baseline, and other trial data, including any related processes to promote data quality (eg, duplicate measurements, training of assessors) and a description of study instruments (eg, questionnaires, laboratory tests) along with their reliability and validity, if known. Reference to where data collection forms can be found, if not in the protocol	_____13________
	18b	Plans to promote participant retention and complete follow-up, including list of any outcome data to be collected for participants who discontinue or deviate from intervention protocols	____16,17______

Data management	19	Plans for data entry, coding, security, and storage, including any related processes to promote data quality (eg, double data entry; range checks for data values). Reference to where details of data management procedures can be found, if not in the protocol	______20_________
Statistical methods	20a	Statistical methods for analysing primary and secondary outcomes. Reference to where other details of the statistical analysis plan can be found, if not in the protocol	______16_________
	20b	Methods for any additional analyses (eg, subgroup and adjusted analyses)	______16_________
	20c	Definition of analysis population relating to protocol non-adherence (eg, as randomised analysis), and any statistical methods to handle missing data (eg, multiple imputation)	______16_________
**Methods: Monitoring**			
Data monitoring	21a	Composition of data monitoring committee (DMC); summary of its role and reporting structure; statement of whether it is independent from the sponsor and competing interests; and reference to where further details about its charter can be found, if not in the protocol. Alternatively, an explanation of why a DMC is not needed	_______19_______
	21b	Description of any interim analyses and stopping guidelines, including who will have access to these interim results and make the final decision to terminate the trial	_______19_______
Harms	22	Plans for collecting, assessing, reporting, and managing solicited and spontaneously reported adverse events and other unintended effects of trial interventions or trial conduct	______17_________
Auditing	23	Frequency and procedures for auditing trial conduct, if any, and whether the process will be independent from investigators and the sponsor	______19_________
**Ethics and dissemination**			
Research ethics approval	24	Plans for seeking research ethics committee/institutional review board (REC/IRB) approval	______17_________
Protocol amendments	25	Plans for communicating important protocol modifications (eg, changes to eligibility criteria, outcomes, analyses) to relevant parties (eg, investigators, REC/IRBs, trial participants, trial registries, journals, regulators)	______19,20______

Consent or assent	26a	Who will obtain informed consent or assent from potential trial participants or authorised surrogates, and how (see Item 32)	_______9_______
	26b	Additional consent provisions for collection and use of participant data and biological specimens in ancillary studies, if applicable	______13_________
Confidentiality	27	How personal information about potential and enrolled participants will be collected, shared, and maintained in order to protect confidentiality before, during, and after the trial	_______19_______
Declaration of interests	28	Financial and other competing interests for principal investigators for the overall trial and each study site	_______23_______
Access to data	29	Statement of who will have access to the final trial dataset, and disclosure of contractual agreements that limit such access for investigators	_______19,23____
Ancillary and post-trial care	30	Provisions, if any, for ancillary and post-trial care, and for compensation to those who suffer harm from trial participation	_______17_______
Dissemination policy	31a	Plans for investigators and sponsor to communicate trial results to participants, healthcare professionals, the public, and other relevant groups (eg, via publication, reporting in results databases, or other data sharing arrangements), including any publication restrictions	______________
	31b	Authorship eligibility guidelines and any intended use of professional writers	_______24_______
	31c	Plans, if any, for granting public access to the full protocol, participant-level dataset, and statistical code	______23________
**Appendices**			
Informed consent materials	32	Model consent form and other related documentation given to participants and authorised surrogates	______yes________
Biological specimens	33	Plans for collection, laboratory evaluation, and storage of biological specimens for genetic or molecular analysis in the current trial and for future use in ancillary studies, if applicable	_______yes_______

*It is strongly recommended that this checklist be read in conjunction with the SPIRIT 2013 Explanation & Elaboration for important clarification on the items. Amendments to the protocol should be tracked and dated. The SPIRIT checklist is copyrighted by the SPIRIT Group under the Creative Commons "Attribution-NonCommercial-NoDerivs 3.0 Unported" license.

BMJ Open

Risperidone versus placebo for aggression following traumatic brain injury: a feasibility randomised controlled trial.

Journal:	*BMJ Open*
Manuscript ID	bmjopen-2019-036300.R1
Article Type:	Original research
Date Submitted by the Author:	06-Apr-2020
Complete List of Authors:	Deb, Shoumitro (Shoumi); Imperial College London, Centre for Psychiatry<br>Aimola, Lina; Imperial College London, Division of Brain Sciences<br>Leeson, Verity; Imperial College London<br>Bodani, Mayur; Kent and Medway NHS and Social Care Partnership Trust<br>Li, Lucia; Imperial College London<br>Weaver, Tim ; Middlesex University<br>Sharp, David; Imperial College London, Division of Brain Sciences<br>Bassett, Paul; Statsconsultancy Ltd,<br>Crawford, Mike; Imperial College London, Psychological Medicine
**Primary Subject Heading**:	Mental health
Secondary Subject Heading:	Mental health, Neurology
Keywords:	PSYCHIATRY, Adult psychiatry < PSYCHIATRY, Impulse control disorders < PSYCHIATRY

SCHOLARONE™
Manuscripts

I, the Submitting Author has the right to grant and does grant on behalf of all authors of the Work (as defined in the below author licence), an exclusive licence and/or a non-exclusive licence for contributions from authors who are: i) UK Crown employees; ii) where BMJ has agreed a CC-BY licence shall apply, and/or iii) in accordance with the terms applicable for US Federal Government officers or employees acting as part of their official duties; on a worldwide, perpetual, irrevocable, royalty-free basis to BMJ Publishing Group Ltd ("BMJ") its licensees and where the relevant Journal is co-owned by BMJ to the co-owners of the Journal, to publish the Work in this journal and any other BMJ products and to exploit all rights, as set out in our licence.

The Submitting Author accepts and understands that any supply made under these terms is made by BMJ to the Submitting Author unless you are acting as an employee on behalf of your employer or a postgraduate student of an affiliated institution which is paying any applicable article publishing charge ("APC") for Open Access articles. Where the Submitting Author wishes to make the Work available on an Open Access basis (and intends to pay the relevant APC), the terms of reuse of such Open Access shall be governed by a Creative Commons licence – details of these licences and which Creative Commons licence will apply to this Work are set out in our licence referred to above.

Other than as permitted in any relevant BMJ Author's Self Archiving Policies, I confirm this Work has not been accepted for publication elsewhere, is not being considered for publication elsewhere and does not duplicate material already published. I confirm all authors consent to publication of this Work and authorise the granting of this licence.

For peer review only

Title

Risperidone versus placebo for aggression following traumatic brain injury: a feasibility randomised controlled trial

Authors

Shoumitro (Shoumi) Deb,[1] Lina Aimola,[1] Verity Leeson,[1] Mayur Bodani,[2] Lucia Li,[1] Tim Weaver,[3] David Sharp,[1] Paul Bassett,[4] Mike J Crawford.[1]

[1]Department of Brain Sciences, Faculty of Medicine, Imperial College London, UK; [2]Kent and Medway NHS and Social Care Partnership NHS Trust, [3]Middlesex University, [4]Stasconsultancy Ltd.

Correspondence to

Professor Shoumitro (Shoumi) Deb; s.deb@imperial.ac.uk

For peer review only

Abstract

Objectives To conduct a feasibility randomised controlled trial of risperidone for the treatment of aggression in adults with traumatic brain injury (TBI).

Design Multi-centre, parallel design, placebo controlled (1:1 ratio) double-blind feasibility trial with an embedded process evaluation. No statistical comparison was performed between the two study groups.

Setting Four neuropsychiatric and neurology outpatient clinics in London and Kent, UK.

Participants Our aim was to recruit 50 TBI patients over 18 months. Follow up participants at 12 weeks using a battery of assessment scales to measure changes in aggressive behaviour and irritability (MOAS-primary outcome, IRQ) as well as global functioning (GOS-E, CGI) and quality of life (EQ-5D-5L, SF-12), mental health (HADS) and medication adverse effects (UKU).

Results Six participants were randomised to the active arm of the trial and eight to the placebo arm over a 10-month period (28% of our target). Two participants withdrew because of adverse events. Twelve out of 14 (85.7%) patients completed a follow up assessment at 12 weeks. At follow up, the scores of all outcome measures improved in both

groups. Placebo group showed numerically better score change according to the primary outcome MOAS. No severe adverse events were reported. The overall rate of adverse events remained low. Data from the process evaluation suggest that existence of specialised TBI Follow-up clinics, availability of a dedicated database of TBI patients' clinical details, simple study procedures and regular support to participants would enhance recruitment and retention in the trial. Feedback from participants showed that once in the study, they did not find the trial procedure onerous.

Conclusions It was not feasible to conduct a successful randomised trial of risperidone versus placebo for post-TBI aggression using the methods we deployed in this study. It is not possible to draw any definitive conclusion about risperidone's efficacy from such a small trial.

Trial registration number: ISRCTN30191436; 19th December 2016. **EudraCT no.** 2015-000641-23.

Strengths and limitations of this study

- We used a stringent RCT methodology for a feasibility trial.
- Once recruited in the study, the participants felt supported.
- A process evaluation helped to identify factors that may support or impede successful recruitment in future trials of interventions for post-TBI aggression.
- Recruitment has remained a major problem.
- Regulatory authority delays hampered patient recruitment.

Introduction

A high proportion of traumatic brain injury (TBI) patients show aggression after the injury (see Table 1). Aggression causes distress to patients and their families, can hinder rehabilitation, lead to social isolation, loss of family/ friends' support, unnecessary use of restraint and medication, and hospitalization. In addition, it has financial implications for patients, their families and society as a whole.

The rates of post-TBI aggression vary widely (see Table 1) depending on the population studied, definition used for aggression, time after injury, severity of injury and presence of comorbidities. There is no universally accepted definition of aggression, therefore, researchers have used different arbitrary cut off scores using different rating scales to define aggression which produced a wide range of incidence rates. Also, the boundary between aggression, agitation, irritability, anger and disinhibition is not clear as different studies

For peer review only - http://bmjopen.bmj.com/site/about/guidelines.xhtml

included different symptoms for assessment. The population point prevalence of post-TBI aggression is unclear. Most studies included patients from clinics or rehabilitation centres, therefore, it is difficult to know the true extent of the problem within the TBI population in general as most of them suffer from a mild TBI (70%-85%)[1] and do not attend any clinic or rehabilitation centre. The rate also varies depending on patient or informant rating as informants tend to report a higher rate of aggression than the patients themselves.[2]

Post-TBI aggression is mostly reactive in nature and not pre-meditated. Verbal aggression and aggression to objects are more common than physical aggression to others.[3]

It is proposed that in the period immediately following TBI, aggression is caused by organic factors such as post-TBI confusion, frontal lobe damage, left cerebral lesion etc.[4] However, in the chronic stage, psychosocial factors play a more important role in causing aggression.[5,6] Many pre and post injury factors affect the rate and the severity of aggression. For example, pre-morbid factors such as antisocial personality trait including aggressive tendency, history of psychiatric disorders, impulsivity, alcohol and substance abuse, low educational and socioeconomic status are shown to predispose aggression after TBI.[5,6] Similarly, post-TBI factors such as loss of job, alcohol and substance abuse, co-morbid anxiety and depression are shown to be associated with a higher rate of post-TBI aggression.[5,6]

Neural substrate of aggression

It is postulated that either a reduction in the top-down control from the pre-frontal cortex or an overactive limbic system (particularly affecting anterior cingulate gyrus and amygdala) may lead to aggression. These regions are responsible for cognitive interpretation of emotional stimuli which determines a person's response to potentially threatening stimuli. It has been suggested that reduced serotonin and increased dopamine and noradrenaline activity in cortical areas, and reduced GABA and acetylcholine activity in limbic areas modulate aggression in humans.[7] Therefore, we can expect that a reduction in cortical dopamine by antipsychotics, nor-adrenaline by beta-blockers and increase in serotonin by selective serotonin reuptake inhibitors, and stabilization of GABA/Glutamate imbalance in the limbic region by anti-epileptics should lead to reduced aggression. However, randomized control trial (RCT) based evidence does not support this simple prediction (see Table 2). It could be because the role of any particular neuromodulator in aggression is far from clear and they may cause aggression through indirect mechanisms. For example, serotonin is involved in influencing impulsivity and dopamine is responsible for reward punishment behaviour rather than aggression per se, and noradrenaline acts as a stress hormone and influences the 'fight-or-flight' response.[8]

For peer review only - http://bmjopen.bmj.com/site/about/guidelines.xhtml

Evidence on pharmacological intervention for post-TBI aggression

Since Deb and Crownshaw's[9] original systematic review on the effectiveness of psychotropics on neurobehavioral symptoms of TBI, several reviews have been published on this.[10-19] Findings from all the RCTs on pharmacotherapy for post-TBI aggression are summarized in Table 2. A number of studies have shown methylphenidate's (MPH) effectiveness in improving post-TBI cognitive impairment and sometimes apathy symptoms.[14] However, only two very small trials (see Table 2) have specifically looked at its effect on post-TBI anger and aggression, one showed superiority of MPH and the other did not.

There are four RCTs involving amantadine, three of which are from the same author (see Table 2). Two of the studies used Neuropsychiatric Inventory-Irritability (NPI-I) Scale, which measures irritability rather than aggression.[20] NPI is not an ideal instrument to assess post-TBI aggression as it was originally designed for dementia patients. Also, half of the 8 items in NPI-A (NPI-Aggression) subscale do not directly relate to aggression. Most studies showed large placebo effect. Overall finding from these studies is equivocal. One other very small (n=10) cross over study of amantadine that was reported by a different group did not show any significant intergroup difference in outcome. There is now a major concern about dopaminergic drugs' serious adverse effects such as impulse control disorder like pathological gambling, hypersexuality and binge eating.[21]

There are four very small crossover trials on beta-blockers, three of which were reported by the same group (see Table 2). Three RCTs used crossover design and one parallel design. Two studies showed superiority of beta-blockers, one was non-significant, and one showed partial superiority. These studies have not always used standardized outcome measures and sample sizes are very small. Additionally, both the treatment period and the follow up are of short duration. Therefore, it is difficult to draw any definite conclusion about beta-blockers' efficacy in treating post-TBI aggression. Furthermore, most studies used a very high dose of beta-blockers which has the potential to cause severe adverse effects such as bronchospasm and hypotension.

Evidence on psychopharmacological intervention of aggression in non-TBI population

As there is no strong evidence to support any specific pharmacological intervention to treat post-TBI aggression, we extrapolated data from RCTs among other patient groups. Old generation antipsychotics by blocking non-specific dopaminergic activity (particularly involving the nigrostriatal pathway) and also for their anti-cholinergic effect cause troublesome adverse effects such as extrapyramidal and cardiac symptoms and also show a higher tendency to develop serious adverse effects such as neuroleptic malignant syndrome. On the other hand, new generation antipsychotics provide better tolerability and a broader therapeutic effect by

For peer review only - http://bmjopen.bmj.com/site/about/guidelines.xhtml

specific action on the D2 dopaminergic system (particularly involving the meso-cortical pathway) as well as serotonergic and nor-adrenergic systems. However, they are prone to produce metabolic syndrome. Therefore, as expected most evidence is based on new generation antipsychotics especially risperidone.

There are six placebo controlled RCTs involving children with autism spectrum disorder and/or intellectual disabilities (ID), all showed superiority of risperidone over placebo (number needed to treat is 3).[22] There are three small RCTs among adults with ID, two showed superiority of risperidone (0.5 mg-4 mgs/day) and one showed no significant intergroup difference among risperidone, haloperidol and placebo.[23] There are two large RCTs both showing superiority of risperidone over placebo in treating aggression in patients with schizophrenia.[24] There are seven RCTs of risperidone (0.5 mg-2 mg/ day) showing its superiority over placebo in treating behaviour and psychiatric symptoms of dementia (BPSD), five of them showed specific effect on aggression.[25] However, because of a high rate of cerebrovascular mortality and extrapyramidal adverse effects associated with antipsychotics these drugs are not recommended for use for BPSD. Some preliminary evidence also exists in favour of olanzapine, aripiprazole and clozapine in treating aggression in different patient groups which we have not discussed here because of lack of space.[26] At present no strong evidence exists in support of other psychotropics such as mood stabilisers, antidepressants, benzodiazepines etc.[26 27]

Justification for our study design

As there is no strong evidence to support any specific drug for the treatment of aggression in TBI, but evidence shows that risperidone may reduce aggression among people with other neuropsychiatric conditions, we set out to examine the feasibility of conducting a placebo controlled trial of risperidone among adults who show aggression following TBI.

Aim

1. To assess feasibility of a future definitive placebo controlled RCT of risperidone for post-TBI aggression in adults.
2. Perform a sample size calculation needed for a future definitive RCT.
3. Through process evaluation identify factors that may support or impede successful recruitment in future trials.

Methods

We conducted a multi-centre parallel design placebo controlled (1:1 ratio) double-blind feasibility RCT of risperidone with an embedded process evaluation.

For peer review only - http://bmjopen.bmj.com/site/about/guidelines.xhtml

We have summarised the design in this paper. For a full description please see our trial protocol paper.[28] Aggression was defined as "any form of behavior directed toward the goal of harming or injuring another living being who is motivated to avoid such treatment,"[29] and included verbal aggression, and physical aggression to others, property and self, excluding agitation. Rather than using an arbitrary cut off score on a rating scale, we asked clinicians to refer any patient to the trial that they were considering prescribing medication for the treatment of post-TBI aggression.

Patient and Public involvement

The study was developed jointly with a group of 18 adults with TBI from a Headway centre in London, UK. They were not participants of the study. A patient with TBI was part of the project group. An advisory group of TBI patients who did not take part in the study met every few months to discuss the project and provide feedback through a facilitator (clinical psychologist). They attended a day centre which was not associated to any of the recruiting centres.

Recruitment

Our aim was to recruit from four centres in London, UK. These included adults with TBI who attended outpatient clinics, three saw patients with various neuropsychiatric disorders including TBI patients and one was a neurology clinic dedicated to TBI adult patients only. The researcher completed a baseline pre-randomisation outcome assessment on those patients who agreed to take part. A local clinician completed the Clinical Global Improvement-Severity (CGI-S) scale[30] and UKU adverse effect scale[31] and arranged for appropriate physical examination. Relevant blood test results were supposed to be collected from participant's case notes if available.

Sample size

As this was a feasibility study, we did not carry out a formal power calculation but estimated that 40-50 is a reasonable number for a feasibility study which would allow us to carry out a sample size calculation for the full future proposed RCT.[32] A general guide for number of participants necessary for a feasibility study is 30 to 50 patients.[33]

Inclusion/ exclusion criteria

Inclusion criteria:

a) aged between 18 and 65 years

For peer review only

For peer review only - http://bmjopen.bmj.com/site/about/guidelines.xhtml

b) patients with a confirmed clinical diagnosis of TBI using the Mayo Clinic criteria,[34] which happened at least six months prior to recruitment
c) referred to the clinicians for the management of aggression and for whom the clinician is considering a pharmacological intervention for aggression after investigating and addressing physical, psychological and social triggers.

Exclusion criteria:

a) patient suffering from Post-Traumatic Amnesia (PTA)
b) co-morbid serious mental illness such as schizophrenia and other psychoses, bipolar disorder, major depressive disorder, personality disorder and dementia
c) patients who were already on an antipsychotic drug or any other drug that may interact with risperidone at the time of randomisation
d) any other contraindication for using risperidone including a previous history of severe adverse events
e) patients with no fixed abode or any other reason for which compliance with study medication and monitoring could pose a major problem
f) a history of definite neurogenic seizures within the last three months.

Active drug dose

We used a flexible dose of risperidone. We started with 1 mg once daily dose and increased the dose if necessary, to up to 4 mgs/day. Equivalent number of placebo capsules were also administered to the appropriate participants. The placebo capsules looked exactly the same as the risperidone capsules. The researcher followed up participants by telephone every week to assess improvement or emergence of any adverse events using informally a scale similar to CGI.[30] The researcher liaised with the local clinician every week to provide feedback on the participant's progress. The local clinicians decided whether a dose increase or decrease is warranted based on the information provided by the researcher and where necessary by personally reviewing the participant in their clinic. Local clinicians were allowed to use PRN (as required) medication if necessary. The researcher provided participants with a proforma to keep a log of medication intake. The researcher checked this during the weekly telephone follow up. At the end of the trial the researcher counted the number of any leftover medicine to assess adherence.

Outcome measures

Feasibility outcome measures

For peer review only - http://bmjopen.bmj.com/site/about/guidelines.xhtml

Our criteria for demonstrating success of the feasibility study were: (a) recruitment of at least 80% of the sample who met inclusion criteria after screening, and (b) completion of follow up assessments at 12 weeks by at least 75% of the randomised participants.

Clinical outcome measures

Primary outcome measure:

We used Modified Overt Aggression Scale (MOAS) to assess improvement in aggression. The MOAS is a simple and widely used 4-item scale that measures verbal aggression along with physical aggression towards other people, property and self. The MOAS[35] is a valid and reliable instrument which is validated in the TBI population[36] and has been used widely in the assessment of post-TBI aggression and its treatment (see Table 1 and 2). Lower MOAS values indicate lower levels of aggression.

Secondary outcome measures:

1) Glasgow Outcome Scale-Extended version (GOS-E),[37] which is a widely used global outcome measure of post-TBI functioning,
2) Irritability Questionnaire (IRQ),[38] (patient and carer version), this is a well validated scale and is used in this trial as irritability is often associated with aggression.
3) Hospital Anxiety and Depression Scale (HADS),[39] which is a well standardised easy to use scale for the assessment of depressive and anxiety symptoms. We used HADS as both anxiety and depressive symptoms are common after TBI and we have established its cut off score for brain injury patients.[40]
4) CGI-I (CGI-Improvement) and CGI-S (CGI-Severity) Scales[30] which are well validated and are used widely as secondary outcome measures in clinical trials,
5) Udvalg for Kliniske Undersøgelser (UKU) Scale[31] to assess adverse effects of risperidone, and
6) Two widely used quality of life (QoL) measures, namely EQ-5D-5L[41] and SF-12[42] to assess participant's quality of life as the ultimate goal of the intervention is not only symptoms reduction but to improve participant's quality of life. These two measures would be useful in calculating Quality Adjusted Life Years (QALYs) in any future definitive RCT.

All these measures were used at baseline and at 12 weeks follow up.

For peer review only

For peer review only - http://bmjopen.bmj.com/site/about/guidelines.xhtml

We used a proforma specifically designed for this study to collect demographic data such as age, gender and also other relevant information such as cause and severity of TBI etc.

Researcher completed MOAS based on information from both the patients and the carers

(where available).

Concealment and randomisation

Remote web-based randomisation was undertaken through a fully automated service operated by an external organisation 'Sealed Envelope'. Our aim was to randomise an equal number of participants to risperidone and placebo. We used random permuted blocks stratified by study centres (n=4). Sealed Envelope designed the randomisation list and labelled all medicine bottles including placebo with randomisation codes before sending to study centres. There was a 24 hours telephone number available for the clinicians and research team if the randomisation code needed to be broken. Everyone involved in the study including the participants, their carers, local clinicians, researchers, general practitioners were blind to this allocation.

Statistical analyses of clinical data

The primary outcome was the patient reported MOAS score, measured at 12 weeks follow up. As this is a feasibility study, no formal hypothesis tests were performed to statistically compare the two study groups, but descriptive data are presented.

All secondary outcomes were analysed descriptively, with no formal hypothesis tests performed. The frequency of adverse events as per the UKU Scale was calculated at follow up.

Process evaluation

A process evaluation using a qualitative method examined trial recruitment, and the feasibility and acceptability of trial procedures from the perspective of patients and their carers. We undertook semi-structured qualitative interviews with trial participants, and their carers, purposively sampled to represent site and treatment allocation.

Interviews were conducted using a bespoke topic guide. As the qualitative interviews generated a rich dataset it is beyond the scope of this paper to present all the details here. The topic guide was developed under the following broad headings; (a) Background information (how TBI has affected participants' lives in general), (b) Interventions (was it difficult to comply with the intervention?), (c) Outcome (how did they feel about the overall

For peer review only - http://bmjopen.bmj.com/site/about/guidelines.xhtml

outcome measures at the end of the study?), (d) Trial procedures (taking part in questionnaire completion, support from the research team etc.), (e) Factors influencing participation and retention (e.g., factors hampering vs. helping with participation). These interviews were audio-recorded, transcribed and analysed using a thematic framework approach[43] and managed using NVivo[44] computer software.

The study received ethical approval from the London-Westminster Research Ethics Committee on behalf of the UK NHS Health Research Authority.

Results

The demographic characteristics of study participants are summarised in Table 3. The mean age of patients was 43 and 39 in the placebo and risperidone groups respectively. Over 60% of both groups were male. Fourteen of the 43 (32.6%) eligible patients were recruited between March 2017 and January 2018 (see Figure 1). Eight patients received placebo and six risperidone (see Figure 1). However, two participants from the placebo group and one from the risperidone group withdrew. Two withdrew due to adverse events and for one participant follow up data were available. Therefore, MOAS data were calculated for five risperidone and seven placebo group participants (see Table 4), giving an 85.7% follow up rate. Verbal aggression was the most common type of aggression exhibited by the participants along with aggression to property, but a minority also showed aggression directed towards self and others. Three participants received 2 mgs and two received 4 mgs risperidone at the end point. No post-intervention blood test results were available from participants' case notes. No one had a psychiatric diagnosis such as schizophrenia, major depressive disorders, anxiety disorders, personality disorder or dementia. No one received concomitant psychotropic medication or PRN (as required) medication.

Feasibility outcome data

Our original recruitment target was 50 participants from four centres over 18 months (0.69 patient/ centre/ month). One centre did not recruit any patient and another centre recruited only one patient who did not complete the trial. The overall recruitment rate was 0.3 patient/ centre/ month across all four centres (21% of the target rate) over 10 months period. The recruitment rate from a research active University centre was 0.78/ month (113% of the target rate). This centre was dedicated to adults with TBI and held a patient database which helped the researcher to screen for potential recruits. Because of regulatory authority delays the total recruitment time available was shortened by 8 months.

Quantitative outcome data

For peer review only - http://bmjopen.bmj.com/site/about/guidelines.xhtml

The data suggest slightly higher MOAS values at baseline in the placebo group. At 12 weeks, MOAS values had reduced in both groups, to a similar level (see Tables 4, 5). Therefore, the placebo group showed a larger change in total MOAS values and its sub-scores. In order to aid a possible future sample size calculation (using an ANCOVA approach), the association between MOAS total score at baseline and 12 weeks was assessed. Spearman's rank correlation gave a correlation of 0.04 between timepoints. This suggests little relationship between the scores at the two timepoints.

The secondary outcome measures showed mixed results (see Table 6). Numerically the score changes in the IRQ and HADS-Anxiety were slightly greater in the risperidone group whereas HADS-Depression score change was slightly greater in the placebo group. A similar picture was revealed for the QoL scales scores (SF-12 and EQ-5D-5L), and also GOS-E (see Table 6). According to CGI-I, 40% in the risperidone group and 33% in the placebo group respectively were 'much' or 'very much improved' at follow up. Patient MOAS score correlated with both baseline (r: 0.78; p<0.001) and follow up patient IRQ severity score (r: 0.64; p=0.03).

No severe adverse events were reported. The overall rate of adverse events remained low although it was slightly higher numerically in the risperidone group.

An approximate sample size calculation done by the study statistician (PB) shows that for a 5% significance level and 90% power, 72 patients per group and 144 in total will be required. Allowing for a 20% attrition rate, a total of 180 patients would need to be recruited.

Qualitative outcome data

Eight patients and two of their carers were interviewed and the data were analysed by the researcher (LA), under the supervision from the qualitative investigator (TW).

Key factors exerting a positive influence on participation reported by patients and their carers included a belief in the positive benefit of research. This may have been expressed as (a) a perception that the trial may help to achieve their 'desire to get better', or (b) satisfy an altruistic motivation to participate in research that might help other people in a similar situation. Some also reported (c) previous positive experiences in taking part in research. Also, significant to maintaining engagement were (d) the straightforward research procedures, and (e) the high level of trust and support engendered by weekly telephone contact from the researcher (LA) and (f) good relations with the referring doctor. Participation was also associated with (g) a higher educational level of the participants.

The analysis showed that the key reasons for patients not taking part in the study were; (a) a reluctance to change treatment, (b) perceived adverse effects of the study medication, (c) lack of understanding of the reason why the study is undertaken as this was not clearly explained to them, (d) lack of insight into their own condition, so the potential participants did

For peer review only - http://bmjopen.bmj.com/site/about/guidelines.xhtml

not see their behaviour as a problem needing any intervention (e) unwillingness to engage with treatment regime or trial procedures and (f) objection to randomisation manifested as a reluctance particularly on the part of the carers regarding potential allocation into the placebo arm which they thought might make the patient's behaviour worse and difficult to manage.

Furthermore, many participants, particularly those who had memory and attention problem, found it tiring to complete so many assessment measures some of which were quite long. Similarly, memory problems may have affected the consent process thus making some potential patients ineligible for recruitment.

Discussion

Given the very small sample size it is not possible to draw any definite conclusion from the interpretation of the outcome data.

Clinical findings

In the current study risperidone did not show superiority over placebo at follow up as measured using total MOAS score (the primary outcome). No clear pattern emerged in secondary outcomes. Like previous studies most of the aggression in our study was primarily verbal in nature.[3] Also, as expected, there was a correlation between patient reported MOAS and IRQ scores suggesting a clinical association between irritability and aggression. Carer reported mean MOAS values are numerically higher than the patient reported values both at baseline and at follow up. This is in keeping with previous studies[3] but difficult to comment on given the small sample size and skewed distribution of MOAS values. This could be due to patient's poor insight to their problems, using denial as a psychological defence and impaired self-awareness.[3]

Risperidone dosage

There was no evidence of non-adherence to medication in this trial. It is difficult to determine the right therapeutic window for the risperidone dosage as a lower dose may be ineffective and a higher dose may produce unwanted adverse effects. This is even more difficult to decide for TBI patients as there is no research-based evidence to calibrate the dose specifically for this patient group. Therefore, we had to look into dosage used in RCTs for non-TBI patient groups for a reference point. We used a lower dose of risperidone than that used for the treatment of schizophrenia for the following reasons; (a) a high dose of risperidone is likely to lower seizure threshold and the TBI patients are vulnerable to develop seizures, we, therefore started low and went slow, (b) reports from one laboratory have shown detrimental effect on experimentally induced TBI rodents' behaviour and cognition from chronic use of

For peer review only - http://bmjopen.bmj.com/site/about/guidelines.xhtml

For peer review only

high dose risperidone, although this finding has not been replicated in humans[45] (c) RCTs of risperidone among adults with ID[23] and also dementia[25] used a lower dose (2mgs-4 mgs/day), (d) in the UK clinicians use low dose risperidone to treat post-TBI aggression (0.5 mgs-4 mgs/day), (e) we were advised by the patient advisory group to use low dose as they were worried about risperidone's adverse effects at a high dose. Also, it appears that a low dose (2 mgs/day) was sufficient for most participants in our study.

Feasibility outcome

The purpose of the study was to assess feasibility of a future definitive RCT. We concluded that it may not be feasible to conduct a definitive trial using the approach we took in this feasibility study. Our rate of recruitment fell short of our original goal because two of the four centres failed to recruit participants. However, if the rate of recruitment achieved in the two active sites, were replicated across other centres, a definitive RCT would be feasible.

We also concluded that an adequate number of participants could not be recruited in a future definitive full RCT from Neuropsychiatry clinics alone. We believe that to recruit an adequate number of participants in the future, it would be necessary to broaden the range of services to include Neurorehabilitation centres and possibly Trauma centres.

Our experience shows that research active centres are likely to recruit an adequate number of participants in a future RCT as they already have the infrastructure and the patients attending these centres are already familiar with research procedures and may have already taken part in other research studies. However, the downside may be that patients may have already been recruited in one or more studies, therefore, would be difficult to recruit into yet another study.

We found the way the project is presented to the potential participant is of utmost importance. Therefore, we feel that a face to face meeting with the potential patients explaining the study properly than a telephone call is more likely to increase the chance of recruitment. Regular support such as weekly telephone calls (as was the case in our study) is likely to retain the patient in the study.

We found that it is important to carefully choose exclusion criteria as in our study initial criterion of excluding patients with a history of epilepsy meant exclusion of some otherwise eligible patients. We later revised the exclusion criterion to 'history of a definite neurogenic seizure within the last three months.'

Recommendations from the feasibility study

A successful definitive RCT would require: (a) recruitment through specialised TBI clinics, (b) clinics having databases of potentially eligible patients, (c) a senior clinician

For peer review only - http://bmjopen.bmj.com/site/about/guidelines.xhtml

engaged enough to facilitate the study and (d) clinical colleagues who are enthusiastic and prepared to help with the study, (e) regulatory approvals in place well before the planned start of recruitment (f) availability of sufficient resources to be able to spend time with potential recruits in a face to face meeting to describe the study, (g) regular support through either regular visits or telephone checks, (h) simple study procedures to optimise retention in the study, for example, make the study medication easily accessible and keep the number of questionnaires and tests to absolute minimum (i) information in an accessible format as many TBI patients would have difficulty in concentrating and memorising complex information and (j) some form of non-pharmacological intervention such as 'anger management' alongside study medication to increase the recruitment and retention rate.

Strengths and limitations

The RCT design in our study is robust and would create minimum selection, performance, detection, attrition, reporting, concealment and other biases. Once recruited in the study, the participants felt supported. A process evaluation helped to get positive and negative feedbacks from the participants. However, recruitment remained a major problem and delays in obtaining regulatory approvals hampered patient recruitment. The total number of participants was too small. Although we achieved our feasibility target of 75% retention, we failed to recruit at least 80% of the sample who met inclusion criteria after screening.

Figure 1: Flow chart of study recruitment.

Authors' contribution

SD was involved in the conception and design of the study, supervised data collection, and wrote the funding application, the study protocol and this manuscript. LA contributed to study protocol, collected all research data and liaised with recruiting sites, carried out qualitative interviews with patients and their carers, and contributed to this manuscript. VL contributed to study protocol, overseen regulatory procedures including Ethics and MHRA approval and supervised the day to day running of the trial, and contributed to this manuscript. MB contributed to the study protocol, recruited patients and contributed to this manuscript. LL recruited patients and contributed to this manuscript. TW contributed to the study protocol, supervised qualitative interview data analysis, and contributed to this manuscript. DS contributed to study protocol, recruited patients and contributed to this manuscript. PB helped with the study design, developed a statistical analysis plan (SAP) and carried out the statistical analyses, and contributed to this manuscript. MC contributed to the study protocol, supervised

For peer review only - http://bmjopen.bmj.com/site/about/guidelines.xhtml

day to day dealings with the Sponsor and contributed to this manuscript. All authors approved the final version of the manuscript.

Competing interest

None declared.

Funding

This article presents independent research funded by the National Institute for Health Research (NIHR), UK under its Research for Patient Benefit (RfPB) Programme (Grant Reference Number PB-PG-1013-32054). The views expressed are those of the authors and not necessarily those of the NHS, the NIHR or the Department of Health, UK.

Availability of supporting data

Only the designated trial investigators have access to the personal data of the participants and to the final dataset. The original e-CRF pages generated during the study is the property of the sponsor (Central and North West London Partnership NHS Foundation Trust, UK). Regulatory bodies if appropriate may request access to personal data. There may be opportunity to share data anonymously if necessary and appropriate after proper authorisation and approval are obtained.

Ethical approval and consent to participate

An ethical approval and consent to participate were granted by the London and Westminster Research Ethics Committee (REC), UK NHS-Medicine Health Research Authority (MHRA), Research Ethics Service, UK on the 30th December 2015. All participants have provided a written consent before participating in the study.

Ethics and regulatory requirements

The sponsor has ensured that the trial Protocol, Study Information Sheets, Consent Forms, GP letter and submitted supporting documents have been approved by the MHRA and a main REC, prior to any patient recruitment. The protocol and all agreed substantial protocol amendments, were documented and submitted for ethical and, other necessary, regulatory approval prior to implementation according to Sponsor Specific Operational Procedures (SOPs).

Acknowledgement

For peer review only - http://bmjopen.bmj.com/site/about/guidelines.xhtml

The Imperial Biomedical Research Centre Facility which is funded by the National Institute of Health Research, UK provided support for the study. Dr Niruj Agrawal for recruiting participants and participating patients and their carers, and also Clinical Services Officers who helped to collect data. Patient advisory group and their facilitator, Dr Anita Rose.

For peer review only

For peer review only - http://bmjopen.bmj.com/site/about/guidelines.xhtml

References

1. Deb S. ICD-10 codes detect only a proportion of all head injury admissions. *Brain Inj* 1999;13(5):369-73.
2. Sabaz M, Simpson GK, Walker AJ, et al: Prevalence, comorbidities, and correlates of challenging behavior among community-dwelling adults with severe traumatic brain injury: a multicenter study. *J Head Trauma Rehabil* 2014;29:E19-E30.
3. Dyer KF, Bell R, McCann J, et al: Aggression after traumatic brain injury: analyzing socially desirable responses and the nature of aggressive traits. *Brain Inj* 2006;20:1163-73.
4. Tateno A, Jorge RE, Robinson RG. Clinical correlates of aggressive behavior after traumatic brain injury. *J Neuropsychiatry Clin Neurosci* 2003;15:155-60
5. Deb S, Lyons I, Koutzoukis C, et al. Rate of psychiatric illness one year after traumatic brain injury. *American J Psychiatry* 1999;156:374-8.
6. Roy D, Vaishnavi S, Han D, Rao V. Correlates and prevalence of aggression at six months and one year after first-time traumatic brain injury. *J Neuropsychiatry Clin Neurosci* 2017;29:334-42.
7. Deb S, Deb T. Neurobiology of aggression. *Neuropsychiatry News* 2016;12:22-6.
8. Siever LJ. Neurobiology of aggression and violence. *Am J Psychiatry* 2008;165:429-42.
9. Deb S, Crownshaw T. The role of pharmacotherapy in the management of behaviour disorders in traumatic brain injury patients. *Brain Inj* 2004;18(1):1-31.
10. Fleminger S, Greenwood RRJ, Oliver DL. Pharmacological management for agitation and aggression in people with acquired brain injury. Cochrane Database of Systematic Reviews 2006, Issue 4. Art. No.: CD003299. DOI: 10.1002/14651858.CD003299.pub2.
11. Warden DL, Gordon B, McAllister TW, et al. Guidelines for the pharmacologic treatment of neurobehavioral sequelae of traumatic brain injury. *J Neurotrauma* 2006;23(10);1468-501.
12. Sadavoy J, Lanctôt KL, Deb S. Management of behavioural and psychological symptoms of dementia and acquired brain injury. In 'Cambridge Textbook of Effective Treatments in Psychiatry' (Eds. P. Tyrer & K. R. Silk), 2011 Cambridge University Press, Cambridge, UK. P. 187-216.
13. Lu J, Gary KW, Neimeier JP. Randomized controlled trials in adult traumatic brain injury. *Brain Inj* 2012;26(13-14):1523-48.
14. Jenkins PO, Mehta MA, Sharp DJ. Catecholamines and cognition after traumatic brain injury. *Brain* 2016;139; 2345-23.

For peer review only - http://bmjopen.bmj.com/site/about/guidelines.xhtml

15. Luaüte J, Plantier D, Wiart L, et al. Care management of the agitation or aggressiveness crisis in patients with TBI. Systematic review of the literature and practice recommendations. *Annals Phys Rehab Med* 2016;59:58-67.

16. Mehta S, McIntyre A, Janzen S, et al. Pharmacological management of agitation among individuals with moderate to severe acquired brain injury: A systematic review. *Brain Inj* 2018;32:287-96.

17. Kim S, Mortera M, Hu X, et al. Overview of pharmacological interventions after traumatic brain injuries: impact on selected outcomes. *Brain Inj* 2019;33(4):442-55.

18. Williamson D, Frenette AJ, Burry LD, et al. Pharmacological interventions for agitated behaviours in patients with traumatic brain injury: a systematic review. BMJ Open 2019;9:e029604. doi:10.1136/ bmjopen-2019-029604

19. Hicks AJ, Clay FJ, Hopwood M, et al. The efficacy and harms of pharmacological interventions for aggression after traumatic brain injury-systematic review. *Frontiers Neurol* 2019 doi: 10.3389/fneur.2019.01169.

20. Cummings JL. The Neuropsychiatric Inventory: Assessing psychopathology in dementia patients. *Neurology* 1997;48(S: 6):S10-S16.

21. Moore TJ, Glenmullen J, Mattison DR. Reports of pathological gambling, hypersexuality, and compulsive shopping associated with dopamine receptor agonist drugs. *JAMA Int Medicine* 2014;174(12):1930-3.

22. Unwin GL, Deb S. Efficacy of atypical antipsychotic medication in the management of behaviour problems in children with intellectual disabilities and borderline intelligence: a systematic review. *Res Develop Disabil* 2011;32:2121-33.

23. Deb S. Psychopharmacology. In: N. N. Singh (Ed.), 'Handbook of Evidence-Based Practices in Intellectual and Developmental Disabilities, Evidence-Based Practices in Behavioral Health.' 2016 Springer International Publishing, Switzerland, Cham. P. 347-81.

24. Marder SR, Davis JM, Chouinard G. The effects of risperidone on the five dimensions of schizophrenia derived by factor analysis: combined results of the North American Trials. *J Clin Psychiatry* 1997;58:538-46.

25. Ballard CG, Waite J, Birks J. Atypical antipsychotics for aggression and psychosis in Alzheimer's disease (Review). Cochrane Database of Systematic Reviews 2006; Issue 1. Article no. CD003476. Doi: 10.1002/14651858.CD003476.pub2.

26. Deb S, Deb T. Neuropsychiatry of aggression. In *'Oxford Textbook of Neuropsychiatry'* (Eds. R. Faruqui, M Bodani and N. Agrawal). *Oxford University Press* 2020 London, UK. P. 379-91.

For peer review only

For peer review only - http://bmjopen.bmj.com/site/about/guidelines.xhtml

27. Comai S, Tau M, Pavlovic Z. The Psychopharmacology of aggressive behavior: a translational approach Part 2: clinical studies using atypical antipsychotics, anticonvulsants, and lithium. *J Clin Psychopharmacol* 2012;32(2):237-60.
28. Deb S., Leeson V., Aimola L., et al. Aggression Following Traumatic brain injury: Effectiveness of Risperidone (AFTER)-a feasibility RCT: study protocol for a randomised controlled trial. *Trials* 2018. 19:325, P. 1-10. Doi: 10.1186/s13063-018-2601-z
29. Baron RA, Richardson DR. Human aggression, (2nd Edition). New York, NY: 1994 Plenum Press.
30. National Institute of Mental Health. CGI Clinical Global Impression Scale-NIMH. *Psychopharmacol Bulletin* 1985;21:839-844.
31. Lingjaerde O, Ahlfos UG, Bech P, et al. The UKU side effect rating scale: a new comprehensive rating scale for psychotropic drugs, and a cross-sectional study of side effects in neuroleptic-treated patients. *Acta Psychiatrica Scandinavica* 1987;334 (Suppl.):1-100.
32. Lancaster GA, Dodd S, Williamson PR. Design and analysis of pilot studies: recommendations for good practice. *J Evaluation Clin Practice* 2002;10:307-12.
33. Field A. Discovering Statistics using SPSS (2nd Ed.). 2005;London:Sage.
34. Malec JF, Brown AW, Leibson CL, et al. The Mayo classification system for traumatic brain injury severity. *J Neurotrauma* 2007;24:1417-24.
35. Ratey JJ, Gutheil CM. The measurement of aggressive behaviour: reflections on the use of the Overt Aggression Scale and the Modified Overt Aggression Scale. *J Neuropsychiatry* 1991;3:557-60.
36. Alderman N, Knight C, Morgan C. Use of a modified version of the Overt Aggression Scale in the measurement and assessment of aggressive behaviours following brain injury. *Brain Inj* 1997;11:503–523.
37. Wilson JTL, Pettigrew LEL, Teasdale GM. Structured interviews for the Glasgow Outcome Scale and the Extended Glasgow Outcome Scale: Guidelines for their use. *J Neurotrauma* 1998;15(8):573-85.
38. Craig KJ, Heitanen H, Markova IS, Berrios GE. The Irritability Questionnaire: a new scale for the measurement of irritability. *Psychiatry Res* 2008;159:367-75.
39. Zigmond AS, Snaith RP. The Hospital Anxiety and Depression Scale. *Acta Psychiatrica Scandinavica* 1983;67:361-70.
40. Falope ZF, Deb S, Rickards H, Powell T. The validity of Hospital Anxiety and Depression Scale and Beck's Depression Inventory-II in acquired brain injury. *Neurorehabil Neural Repair* 2006;20:90.

For peer review only

For peer review only - http://bmjopen.bmj.com/site/about/guidelines.xhtml

41. Herdman M, Gudex C, Lloyd A, et al. Development and preliminary testing of the new five-level version of EQ-5D (EQ-5D-5L). *Qual Life Res* 2013;20(10):1727-36.

42. Ware JE Jr., Mark K, Susan DK. A 12-Item Short-Form Health Survey: construction of scales and preliminary tests of reliability and validity. *Medical Care* 1996;34(3):220-33.

43. Hesse-Biber S, Leavy P. The practice of qualitative research. Thousand Oaks, CA 2006; Sage Publications.

44. NVivo 12 Plus. QSR International UK Ltd., 2019; London, UK.

45. Kline AE, Massucci JL, Zafonte RD, et al. Differential effects of single versus multiple administrations of haloperidol and risperidone on functional outcome after experimental brain trauma. *Crit Care Med* 2007; 35(3): 919-24.

46. Brooks N, Campsie L, Symington C, et al. The effects of severe head injury on patient and relative within seven years of injury. *J Head Trauma Rehabil* 1987;2(3):1-13.

47. Deb S, Lyons I, Koutzoukis C. Neurobehavioural symptoms one year after a head injury. *British J Psychiatry* 1999;174:360-5.

48. Baguley IJ, Cooper J, Felmingham K. Aggressive behavior following traumatic brain injury: how common is common? *J Head Trauma Rehabil* 2006;21:45-56.

49. Draper K, Ponsford J, Schönberger M. Psychosocial and emotional outcomes 10 years following traumatic brain injury. *J Head Trauma Rehabil* 2007;22:278-87.

50. Kelly G, Brown S, Todd J, Kremer P. Challenging behaviour profiles of people with acquired brain injury living in community settings. *Brain Inj* 2008;22:457-70.

51. Rao V, Rosenberg P, Bertrand M, et al. Aggression after traumatic brain injury: prevalence and correlates. *J Neuropsychiatry Clin Neurosci* 2009;21:420-429.

52. Ciurli P, Formisano R, Bivona U, et al. Neuropsychiatric disorders in persons with severe traumatic brain injury: prevalence, phenomenology, and relationship with demographic, clinical, and functional features. *J Head Trauma Rehabil* 2011;26:116-26.

53. Lange RT, Brickell TA, French LM, et al. Neuropsychological outcome from uncomplicated mild, complicated mild, and moderate traumatic brain injury in US military personnel. *Archives Clin Neuropsychol* 2012;27:480-94.

54. James AI, Young AW. Clinical correlates of verbal aggression, physical aggression and inappropriate sexual behaviour after brain injury. *Brain Inj* 2013;27:1162-72.

55. Mooney GF, Haas LJ. Effect of methylphenidate on brain injury-related anger. *Arch Phys Med Rehabil* 1993;74:153-60.

56. Speech TJ, Rao SM, Osmon DC, Sperry LT. A double-blind controlled study of methylphenidate treatment in closed head injury. *Brain Inj* 1993;7:333-8.

For peer review only

For peer review only - http://bmjopen.bmj.com/site/about/guidelines.xhtml

57. Schneider WN, Drew-Cates J, Wong TM, Dombovy ML. Cognitive and behavioural efficacy of amantadine in acute traumatic brain injury: An initial double-blind placebo-controlled study. *Brain Inj* 1999;13(11):863-72.

58. Hammond FM, Bickett AK, Norton JH, Pershad R. Effectiveness of amantadine hydrochloride in the reduction of chronic traumatic brain injury irritability and aggression. *J Head Trauma Rehabil* 2014;29:391-9.

59. Hammond FM, Sherer M, Malec JF, et al. Amantadine effect on perceptions of irritability after traumatic brain injury: results of the amantadine irritability multisite study. *J Neurotrauma* 2015;32:1230-8.

60. Hammond FM, Malec JF, Zafonte RD, et al. Potential impact of amantadine on aggression in chronic traumatic brain injury. *J Head Trauma Rehabil* 2017;32:308–18.

61. Greendyke RM, Kanter DR, Schuster DB, et al. Propranolol treatment of assaultive patients with organic brain disease. A double-blind crossover, placebo-controlled study. *J Nervous Mental Disease* 1986;174(5):290-4.

62. Greendyke RM, Kanter DR. Therapeutic effects of pindolol on behavioral disturbances associated with organic brain disease: a double-blind study. *J Clin Psychiatry* 1986;47(8):423-6.

63. Greendyke RM, Berkner JP, Webster JC, Gulya A. Treatment of behavioral problems with pindolol. *Psychosomatics* 1989;30(2):161-5.

64. Brooke MM, Patterson DR, Questad KA, et al. The treatment of agitation during initial hospitalization after traumatic brain injury. *Archives Phys Med Rehabil* 1992;73(10):917-21.

Table 1: Rates of post-TBI aggression

Study	Participants	Follow Up	Assessment	Findings
Brooks (1987)[46]	134 severe TBI patients discharged from hospital	7 years	Researcher designed structured interview	74% of relatives' reporting and 62% of patients' reporting of anger/ irritability/ impatience
Deb (1999)[47]	196 TBI patients	12 months	Researcher designed	35.3% patients reported irritability and 15% verbal aggression

For peer review only - http://bmjopen.bmj.com/site/about/guidelines.xhtml

	discharged from hospital		structured interview	
Tateno (2003)[4]	89 TBI vs. 26 non-TBI multi-injury patients	6-months	OAS (researcher determined arbitrary cut off score)	33.7% of TBI and 11.5% of non-TBI patients showed aggression. Association with major depression, frontal lobe lesion, poor pre-morbid social functioning, history of alcohol and substance abuse
Dyer (2006)[3]	24 TBI vs. 21 Spinal Cord Injury (SCI) patients vs. 24 uninjured (UI)	Variable	BPAQ	35%-38% verbal aggression, 25%-39% anger among TBI group. According to informant rating TBI group showed a higher verbal aggression than SCI and UI group but no inter-group difference in physical aggression
Baguley (2006)[48]	228 moderate/ severe TBI patients	Variable	OAS	25% aggressive
Draper (2007)[49]	53 mild to severe TBI patients	10 years	NFI, FSS, SPRS, HADS	12% clinically significant aggression score in NFI-aggression. Association with anxiety, depression, fatigue and alcohol use
Kelly (2008)[50]	190 ABI patients	Variable	OBS	Verbal aggression: 85.8%; Physical aggression to people: 41.1%; to objects: 35.3%; to self: 5.3%
Rao (2009)[51]	67 first time TBI patients	3 months	OAS	28.4% aggressive

For peer review only - http://bmjopen.bmj.com/site/about/guidelines.xhtml

Ciurli (2011)[52]	120 severe TBI patients vs. 77 healthy controls	Variable	NPI	70% verbal aggression and 54% physical aggression to others and objects. TBI group showed a statistically significant higher mean value in NPI-aggression/agitation Scale ($p<0.001$)
Lange (2012)[53]	Military personnel: Uncomplicated (n=24) and complicated mild TBI (n=17)	3-6 months	PAI	Aggression in 45.8% in uncomplicated mild TBI and 23.5% complicated mild TBI group
James, Young (2013)[54]	152 ABI patients	Variable	Researcher designed aggression rating scale	74% verbal aggression and 65% physical aggression
Sabaz (2014)[2]	507 severe TBI patients	Variable	OBS	31.9% aggression
Roy (2017)[6]	103 first time TBI patients	3, 6 and 12 months	OAS	Verbal aggression: 34.3% at 3 months, 41.1% at 6 months and 38% at 12 months. Physical aggression to others: 0% at 3 months, 2.8% at 6 months and 1.4% at 12 months. Aggression to objects: 1.01% at 3 months, 8.2% at 6 months and 7% at 12 months. Self-aggression: 1.02% at 3 months, 5.5% at 6 months and

For peer review only - http://bmjopen.bmj.com/site/about/guidelines.xhtml

				2.8% at 12 months. Association with depression.

ABI: Acquired Brain Injury; BPAQ: Buss-Perry Aggression Questionnaire; FSS: Fatigue Severity Scale; HADS: Hospital Anxiety and Depression Scale; NFI: Neurobehavioral Functional Inventory; NPI: Neuropsychiatry Inventory; OAS: Overt Aggression Scale; OBS: Overt Behaviour Scale; PAI: Personality Assessment Inventory; SPRS: Sydney Psychosocial Reintegration Scale; TBI: Traumatic Brain Injury.

Table 2: RCTs of pharmacological interventions of aggression in post-TBI adults

Author (year)	Drug dose	RCT design	Number of participants	Outcome measure	Findings
**Psychostimulant**					
Mooney (1993)[55]	Methylphenidate 30 mgs daily	Parallel single blind design	Traetment:19 Placebo:19	POMS-Anger hostility score	Significantly effective in anger reduction
Speech (1993)[56]	Methylphenidate 0.3mg/kg twice daily	Cross over	12 closed head injury patients	KAS-belligerence score	NS
**Dopaminergic**					
Schnieder (1999)[57]	Amantadine 100mgs-300mgs/ day	Cross over	10 TBI patients	Neurobehavioral Rating Scale	NS
Hammond (2014)[58]	Amantadine 100 mgs twice daily	Parallel design	76	NPI-I + NPI-A	NS for the whole group but in a subgroup, amantadine was significantly better
Hammond (2015)[59]	Amantadine 100 mgs twice daily	Parallel design	168	NPI-I	NS, both groups showed

For peer review only - http://bmjopen.bmj.com/site/about/guidelines.xhtml

					large improvements
Hammond (2017)[60]	Amantadine 100 mgs twice daily	Parallel design	118	NPI-I	Two items out of many showed significantly better outcome in the amantadine group
**Beta-blockers**					
Greendyke (1986a)[61]	Propranolol 80mgs-520mgs/ day	Cross over	9 ABI patients	Observed frequency of aggressive behaviour	Propranolol significantly reduced assaultive behaviour
Greendyke (1986b)[62]	Pindolol 10mgs-60 mgs/ day	Cross over	11 ABI patients (possible overlap with participants in 1986a study not known)	Observed frequency of aggressive behaviour	Pindolol significantly reduced assaultive behaviour
Greendyke (1989)[63]	Pindolol 5mgs-20 mgs/ day	Cross over	10 ABI patients	OAS	NS
Brooke (1992)[64]	Propranolol 60 mgs-420 mgs/ day (recommended maximum is 320 mgs/ day)	Parallel design	Propranolol:11<br>Placebo:10	OAS	Propranolol group showed less intense but same frequency of assaults to the placebo group,

For peer review only - http://bmjopen.bmj.com/site/about/guidelines.xhtml

					placebo group received more physical restraints

ABI: Acquired Brain Injury; KAS: Katz Adjustment Scale; NPI-I: Neuropsychiatry Inventory-Irritability; NPI-A: NPI-Aggression; NS: statistically non-significant intergroup difference; OAS: Overt Aggression Scale; POMS: Profile of Mood States; TBI: Traumatic Brain Injury.

Table 3: Patient demographics

Variable	Category	Placebo	Risperidone
Number patients	-	8	6
Gender	Female	3 (38%)	1 (17%)
	Male	5 (62%)	5 (83%)
Age	-	43.1 ± 11.3 years	39.3 ± 8.7 years
TBI Cause	Assault	2 (25%)	0 (0%)
	Fall	3 (38%)	1 (17%)
	Road Traffic Accident	3 (38%)	3 (50%)
	Other	0 (0%)	2 (33%)
Initial severity (*)	Mild	2 (33%)	1 (17%)
	Moderate	1 (17%)	3 (50%)
	Severe	3 (50%)	2 (33%)
History of coma	No	5 (62%)	3 (50%)
	Yes	3 (38%)	3 (50%)
History of PTA	No	5 (62%)	2 (33%)
	Yes	3 (38%)	4 (67%)

For peer review only

For peer review only - http://bmjopen.bmj.com/site/about/guidelines.xhtml

Summary statistics are: Mean ± SD or Number (percentage)

(*) Missing data for 2 patients in Placebo group

PTA: Post Traumatic Amnesia

Table 4: Primary outcome MOAS-patient ratings

Timepoint	Group		N	Mean ± SD	Median [IQR]	Range
Baseline (+)	Placebo		7	11.6 ± 9.6	8 [2, 24]	2, 25
	Risperidone		5	5.0 ± 3.0	6 [2, 8]	2, 9
Week 12	Placebo		7	2.0 ± 2.4	1 [0, 3]	0, 7
	Risperidone		5	2.2 ± 3.9	0 [0, 6]	0, 9
Change (*)	Placebo		7	-9.5 ± 10.5	-4 [-23, -1]	-25, -1
	Risperidone		5	-2.8 ± 4.8	-2 [-8, 2]	-9, 3

(+) Summary statistics only for patients with values at 12 weeks

(*) Calculated as value at Week 12 minus value at baseline

Table 5: MOAS subdomain scores

		Placebo		Risperidone	
Outcome	Timepoint	N	Summary	N	Summary
MOAS patient	Baseline (+)	7	4.7 ± 2.4	5	3.4 ± 1.7
- Verbal	Week 12	7	1.4 ± 1.3	5	1.0 ± 1.4
	Change (*)	7	-3.3 ± 2.7	5	-2.4 ± 2.3
MOAS patient	Baseline (+)	7	2.0 ± 2.1	5	0.2 ± 0.4
- Against property	Week 12	7	0.3 ± 0.8	5	0.2 ± 0.4
	Change (*)	7	-1.7 ± 2.4	5	0.0 ± 0.0
MOAS patient	Baseline (+)	7	0.6 ± 0.8	5	0.4 ± 0.9
- Autoaggression	Week 12	7	0.0 ± 0.0	5	0.0 ± 0.0
	Change (*)	7	-0.6 ± 0.8	5	-0.4 ± 0.9
MOAS patient	Baseline (+)	7	0.3 ± 0.8	5	0.0 ± 0.0
- Against people	Week 12	7	0.0 ± 0.0	5	0.2 ± 0.4
	Change (*)	7	-0.3 ± 0.8	5	0.2 ± 0.4

For peer review only

For peer review only - http://bmjopen.bmj.com/site/about/guidelines.xhtml

MOAS carer - Total score	Baseline (+)	5	8.4 ± 8.4	3	10.3 ± 6.5
	Week 12	5	2.8 ± 3.1	3	3.7 ± 3.8
	Change (*)	5	-5.6 ± 5.6	3	-6.7 ± 3.2

(+) Summary statistics only for patients with values at 12 weeks

(*) Calculated as value at Week 12 minus value at baseline

Table 6: Secondary outcome measures (IRQ, GOS-E, EQ-5D-5L, SF-12, HADS)

Outcome	Timepoint	Placebo		Risperidone	
		N	Mean ± SD	N	Mean ± SD
IRQ patient - Severity	Baseline (+)	7	34 ± 6	5	37 ± 19
	Week 12	7	26 ± 7	5	25 ± 14
	Change (*)	7	-8 ± 9	5	-13 ± 13
IRQ carer - Severity	Baseline (+)	5	10 ± 4	5	14 ± 5
	Week 12	5	7 ± 4	5	7 ± 4
	Change (*)	3	-3 ± 2	5	-6 ± 2
GOS-E	Baseline (+)	7	6.4 ± 1.1	5	6.6 ± 1.5
	Week 12	7	6.4 ± 1.1	5	6.8 ± 1.6
	Change (*)	7	0.0 ± 0.0	5	0.2 ± 0.5
EQ VAS	Baseline (+)	7	53 ± 24	5	61 ± 10
	Week 12	7	57 ± 30	5	64 ± 21
	Change (*)	7	4 ± 11	5	3 ± 18
SF-12 - Physical	Baseline (+)	7	44 ± 13	5	51 ± 10
	Week 12	7	43 ± 11	5	47 ± 12
	Change (*)	7	0 ± 14	5	-3 ± 5
SF-12 - Mental	Baseline (+)	7	34 ± 14	5	30 ± 12
	Week 12	7	41 ± 15	5	40 ± 19
	Change (*)	7	7 ± 18	5	9 ± 13
HADS anxiety	Baseline (+)	7	8.6 ± 3.8	5	9.6 ± 6.9
	Week 12	7	6.7 ± 3.8	5	6.8 ± 7.2
	Change (*)	7	-1.9 ± 3.5	5	-2.8 ± 1.8
HADS depression	Baseline (+)	7	9.9 ± 5.5	5	4.8 ± 4.2
	Week 12	7	7.9 ± 4.7	5	5.0 ± 3.9
	Change (*)	7	-2.0 ± 2.8	5	0.2 ± 2.9

(+) Summary statistics only for patients with values at 12 weeks

For peer review only

For peer review only - http://bmjopen.bmj.com/site/about/guidelines.xhtml

(*) Calculated as value at Week 12 minus value at baseline

For peer review only

For peer review only - http://bmjopen.bmj.com/site/about/guidelines.xhtml

Figure 1: Flow chart of study recruitment:

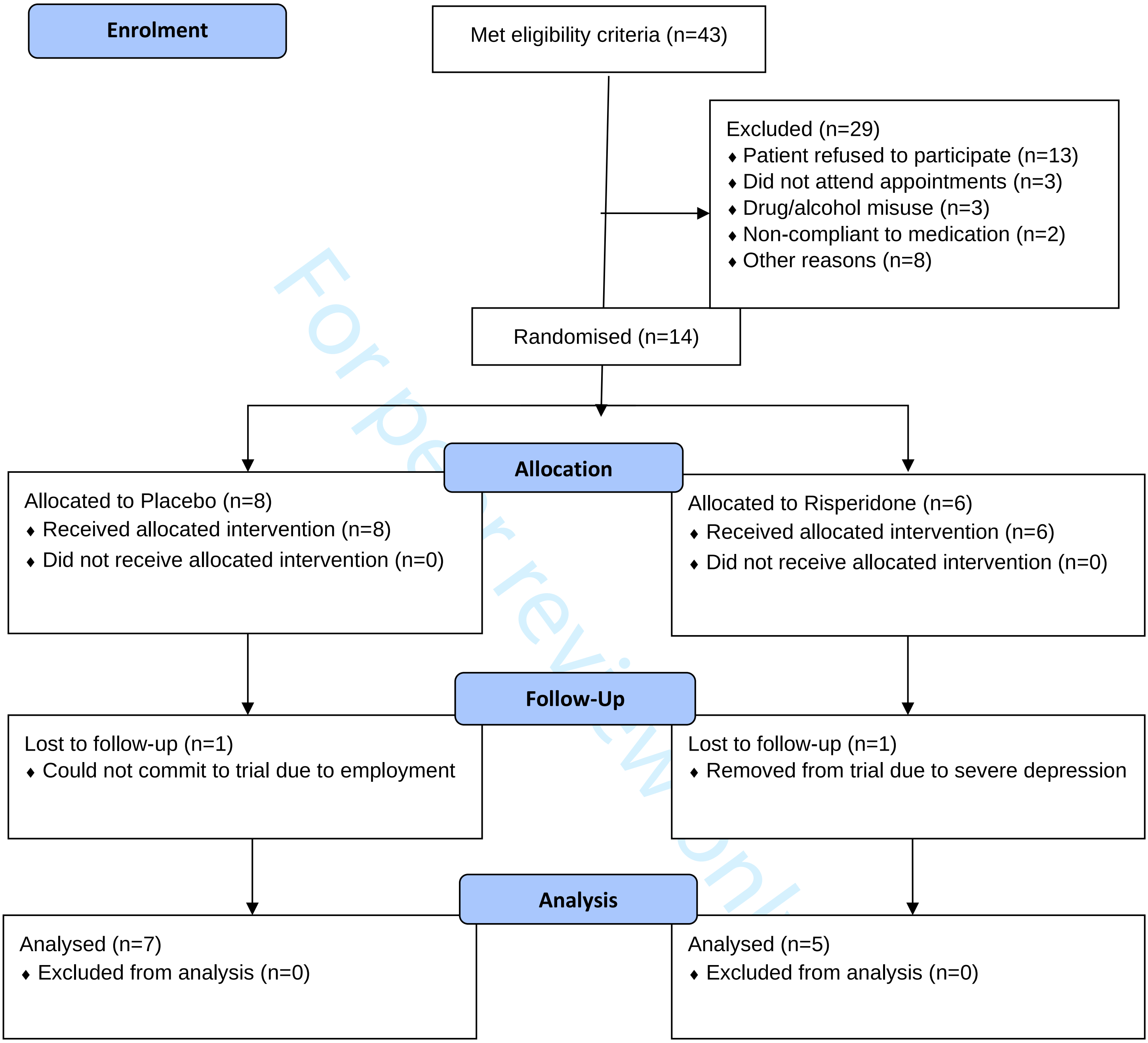

CONSORT 2010 checklist of information to include when reporting a randomised trial*

Section/Topic	Item No	Checklist item	Reported on page No
**Title and abstract**			
	1a	Identification as a randomised trial in the title	1
	1b	Structured summary of trial design, methods, results, and conclusions (for specific guidance see CONSORT for abstracts)	1
**Introduction**			
Background and objectives	2a	Scientific background and explanation of rationale	2,3
	2b	Specific objectives or hypotheses	4
**Methods**			
Trial design	3a	Description of trial design (such as parallel, factorial) including allocation ratio	4
	3b	Important changes to methods after trial commencement (such as eligibility criteria), with reasons	4
Participants	4a	Eligibility criteria for participants	4,5
	4b	Settings and locations where the data were collected	4
Interventions	5	The interventions for each group with sufficient details to allow replication, including how and when they were actually administered	5
Outcomes	6a	Completely defined pre-specified primary and secondary outcome measures, including how and when they were assessed	5
	6b	Any changes to trial outcomes after the trial commenced, with reasons	n/a
Sample size	7a	How sample size was determined	4
	7b	When applicable, explanation of any interim analyses and stopping guidelines	x
Randomisation:			
Sequence generation	8a	Method used to generate the random allocation sequence	6
	8b	Type of randomisation; details of any restriction (such as blocking and block size)	6
Allocation concealment mechanism	9	Mechanism used to implement the random allocation sequence (such as sequentially numbered containers), describing any steps taken to conceal the sequence until interventions were assigned	6
Implementation	10	Who generated the random allocation sequence, who enrolled participants, and who assigned participants to interventions	6
Blinding	11a	If done, who was blinded after assignment to interventions (for example, participants, care providers, those	6

For peer review only

For peer review only - http://bmjopen.bmj.com/site/about/guidelines.xhtml

		assessing outcomes) and how	
	11b	If relevant, description of the similarity of interventions	5
Statistical methods	12a	Statistical methods used to compare groups for primary and secondary outcomes	6
	12b	Methods for additional analyses, such as subgroup analyses and adjusted analyses	6
**Results**			
Participant flow (a diagram is strongly recommended)	13a	For each group, the numbers of participants who were randomly assigned, received intended treatment, and were analysed for the primary outcome	6, Figure1
	13b	For each group, losses and exclusions after randomisation, together with reasons	6, Figure1, Table 3
Recruitment	14a	Dates defining the periods of recruitment and follow-up	6
	14b	Why the trial ended or was stopped	n/a
Baseline data	15	A table showing baseline demographic and clinical characteristics for each group	16, Table 3
Numbers analysed	16	For each group, number of participants (denominator) included in each analysis and whether the analysis was by original assigned groups	6, Figure 1
Outcomes and estimation	17a	For each primary and secondary outcome, results for each group, and the estimated effect size and its precision (such as 95% confidence interval)	6,7 Tables 4,5,6
	17b	For binary outcomes, presentation of both absolute and relative effect sizes is recommended	n/a
Ancillary analyses	18	Results of any other analyses performed, including subgroup analyses and adjusted analyses, distinguishing pre-specified from exploratory	n/a
Harms	19	All important harms or unintended effects in each group (for specific guidance see CONSORT for harms)	7
**Discussion**			
Limitations	20	Trial limitations, addressing sources of potential bias, imprecision, and, if relevant, multiplicity of analyses	9
Generalisability	21	Generalisability (external validity, applicability) of the trial findings	n/a
Interpretation	22	Interpretation consistent with results, balancing benefits and harms, and considering other relevant evidence	8
**Other information**			
Registration	23	Registration number and name of trial registry	1
Protocol	24	Where the full trial protocol can be accessed, if available	9-10
Funding	25	Sources of funding and other support (such as supply of drugs), role of funders	

*We strongly recommend reading this statement in conjunction with the CONSORT 2010 Explanation and Elaboration for important clarifications on all the items. If relevant, we also recommend reading CONSORT extensions for cluster randomised trials, non-inferiority and equivalence trials, non-pharmacological treatments, herbal interventions, and pragmatic trials. Additional extensions are forthcoming: for those and for up to date references relevant to this checklist, see www.consort-statement.org.

For peer review only - http://bmjopen.bmj.com/site/about/guidelines.xhtml

For peer review only
